# Speech, Language and Non‐verbal Communication in CLN2 and CLN3 Batten Disease

**DOI:** 10.1002/jimd.12838

**Published:** 2025-01-16

**Authors:** Lottie D. Morison, Ineka T. Whiteman, Adam P. Vogel, Lisa Tilbrook, Michael C. Fahey, Ruth Braden, Joanna Bredebusch, Michael S. Hildebrand, Ingrid E. Scheffer, Angela T. Morgan

**Affiliations:** ^1^ Speech and Language Murdoch Children's Research Institute Parkville Victoria Australia; ^2^ Department of Audiology and Speech Pathology The University of Melbourne Parkville Victoria Australia; ^3^ Batten Disease Support and Research Association Australia Shelley Beach New South Wales Australia; ^4^ Batten Disease Support, Research and Advocacy Association Columbus Ohio USA; ^5^ Beyond Batten Disease Foundation Austin Texas USA; ^6^ Redenlab Pty Ltd Melbourne Victoria Australia; ^7^ Thrive Health Care Port Pirie South Australia Australia; ^8^ Department of Paediatrics Monash University Clayton Victoria Australia; ^9^ Clinical Sciences Monash Health Clayton Victoria Australia; ^10^ Epilepsy Research Centre The University of Melbourne Heidelberg Victoria Australia; ^11^ Department of Medicine Austin Health Heidelberg Victoria Australia; ^12^ Department of Paediatrics Royal Children's Hospital Parkville Victoria Australia; ^13^ The Florey Institute of Neuroscience and Mental Health Melbourne Victoria Australia; ^14^ Neuroscience Research Group Murdoch Children's Research Institute Melbourne Victoria Australia

**Keywords:** Batten disease, cerliponase alfa, CLN2, CLN3, enzyme replacement therapy, language, neuronal ceroid lipofuscinoses, speech

## Abstract

CLN2 and CLN3 diseases, the most common types of Batten disease (also known as neuronal ceroid lipofuscinosis), are childhood dementias associated with progressive loss of speech, language and feeding skills. Here we delineate speech, language, non‐verbal communication and feeding phenotypes in 33 individuals (19 females) with a median age of 9.5 years (range 3–28 years); 16 had CLN2 and 17 CLN3 disease; 8/15 (53%) participants with CLN2 and 8/17 (47%) participants with CLN3 disease had speech and language impairments prior to genetic diagnosis. At the time of study all participants, bar one, had language impairments. The remaining participant with typical language was tested at age 3 years, following pre‐symptomatic enzyme replacement therapy (ERT) from age 9 months. CLN2 and CLN3 disease had different profiles. For CLN2 disease, all affected individuals showed language impairment with dysarthria; older individuals with classical disease progressively became non‐verbal. For CLN3 disease, the presentation was more heterogeneous. Speech impairment was evident early in the disease course, with dysarthria (13/15, 87%), often manifesting as neurogenic stuttering (5/15, 33%). Participants with CLN2 disease had comparable expressive and receptive language skills (*p* > 0.99), yet participants with CLN3 disease had stronger expressive language than receptive language skills (*p* = 0.004). Speech, cognitive and language impairment and adaptive behaviour showed progressive decline in both diseases. Individuals with pre‐symptomatic ERT or atypical CLN2 disease were less impaired. Challenging behaviours were common in CLN3 (11/17, 65%), but less frequent in CLN2 (4/16, 25%) disease. Individuals with Batten disease require tailored speech therapy incorporating communication partner training utilising environment adaptations and informal communication behaviours.

## 1 Introduction

CLN2 (OMIM 204500) and CLN3 (OMIM 204200) diseases are the most common types of Batten disease, also known as neuronal ceroid lipofuscinoses (NCLs) [1]. CLN2 and CLN3 diseases are autosomal recessive, progressive lysosomal disorders causing dementia in childhood and death by adolescence and the twenties, respectively [2, 3]. Characterisation of communication deficits in individuals with CLN2 and CLN3 diseases has been limited, despite speech and language decline closely mirroring disease progression [4, 5, 6, 7, 8].

CLN2 disease is caused by homozygous or compound heterozygous pathogenic variants in *TPP1* which encodes the lysosomal enzyme tripeptidyl peptidase (HGNC 2073) [9]. CLN2 disease causes late infantile‐onset Batten disease with onset around 2–4 years [4, 10, 11]. Speech and language delay are often the first symptoms of classical CLN2 disease (Table S1) [12, 13, 14, 15]. Most children typically say their first words on time at around 12 months, but combining two words together is typically delayed and some never achieve this milestone [12, 13, 14, 15]. Individuals with classical CLN2 disease have progressive speech, language and visual impairment, cognitive decline, seizures, ataxia and dysphagia [4]. The development of precision medicine for CLN2 disease has been transformational, with enzyme replacement therapy (ERT) slowing disease progression [16, 17, 18]. Without treatment, children typically lose speech by 5 years of age and die by early adolescence [4, 10]. Atypical CLN2 disease deviates with a delayed onset and slower disease course [19, 20].

CLN3 disease causes juvenile‐onset Batten disease and is caused by homozygous or compound heterozygous variants in *CLN3* which encodes a multipass transmembrane protein (HGNC 2074) [21, 22, 23, 24, 25]. CLN3 disease is characterised by visual impairment around 5–6 years of age, followed by a heterogenous disease course including speech, language, cognitive and motor impairment, and seizures, and ultimately premature death [24, 26, 27]. Visual impairment is usually the first symptom, though some children present with communication and cognitive decline [28]. By 10 years of age, cognitive and language decline is evident, alongside dysarthria and dysfluent speech [5, 6, 8, 24, 29, 30, 31].

In CLN2 disease, ERT efficacy was determined using four‐point language and motor clinician‐reported scales to ascertain functional change [16, 32, 33]. Speech and language scales have also captured disease progression in CLN3 disease [25, 34, 35]. Clinician‐administered assessments have captured verbal skills in the early stages of CLN3 disease, but in later stages, subject cooperation with assessments becomes increasingly challenging [6, 8, 24]. Speech and language skills have not been characterised in CLN2 or CLN3 disease across the disease course, beyond the use of disease‐specific scales. Hence, there is limited evidence to inform speech and language interventions in CLN2 and CLN3 disease, though speech therapy is routinely recommended [12, 29, 31, 36, 37].

Precise speech, language and feeding phenotyping in CLN2 and CLN3 disease will characterise disease features at different ages, to map out progression over time. This will inform how to measure and monitor speech and language changes in individuals as their disease progresses, provide targeted speech and language intervention, and evaluate changes in response to novel therapies, such as ERT and gene therapies.

## 2 Methods

### 2.1 Participants

Participants were recruited via flyer advertisement to English‐speaking countries with Batten disease support and research organisations: Australia, New Zealand, Canada, United Kingdom and United States. Participants were aged over 6 months and had homozygous or compound heterozygous pathogenic or likely pathogenic variants in *TPP1* or *CLN3*. Parents provided a clinical genetic testing report to verify a child's diagnosis. Bereaved families whose children had passed away within 2 years of enrolment were also included.

### 2.2 Medical

Parents provided medical and developmental milestone data using a validated questionnaire, and reports from their child's treating clinicians. Bereaved families provided data based on their most recent memories of their child. Parents' reports were further verified by a speech pathologist over a video call. To minimise impact of technological and connection difficulties, the speech pathologist selected was experienced in video call assessments and followed methodology used in previous studies [38, 39, 40]. Bereaved families provided videos of their deceased child.

#### 2.2.1 Feeding

The Child Oral Motor Proficiency Scale (ChOMPS) assessed feeding in participants without enteral feeding [41]. The ChOMPS is a parent questionnaire with normative data from 6 months to 7 years. For participants older than 7 years, the ChOMPS was scored using 7‐year‐old normative data. The Paediatric Assessment Scale for Severe Feeding Problems (PASSFP) assessed participants with enteral feeding [42]. The PASSFP is a 15‐item parent questionnaire with scores ranging from 0 to 66. Low scores on the PASSFP and ChOMPS indicate more feeding difficulties. The Drooling Impact Scale assessed drooling impact, with higher scores reflecting more drooling [43].

### 2.3 Communication

Parents reported changes they had observed in their child's speech and language skills. The Roadmap to Communicative Competence (ROCC) assessed an individual's ability to use language to interact with communication partners across settings, evaluating 10 domains, including symbolic communication, social interaction and communication initiation [44].

#### 2.3.1 Speech

A speech pathologist assessed verbal participants' speech over video call, using a 5‐min conversation sample, syllable repetition diadochokinetic tasks and single word stimuli to rate dysarthric features across the speech subsystems of respiration, phonation, resonance, articulation and prosody [45]. An oral motor assessment also contributed to the differential diagnosis of dysarthria features. Articulation and phonological impairments were also identified [46].

Syllable repetition diadochokinetic tasks (alternating motion rate “papapa” and sequential motion rate “pataka”), counting 1–10, and sustained vowel (/ɐ:/) were acoustically analysed using Redenlab Pty Ltd. software (Table S2) [47]. Quantitative, objective acoustic measurements assessed perturbations in speech subsystems. To identify aberrant speech features, a control group was used with two age‐ and sex‐matched control participants for every participant with Batten disease with acoustic data.

Lastly, the Intelligibility in Context Scale was used to rate speech intelligibility from 1 (never understood) to 5 (always understood) by communication partners (e.g. family, friends, strangers) [48].

#### 2.3.2 Language

The Vineland Adaptive Behaviour Scales Third Edition (Vineland‐3) comprehensive caregiver form assessed language via the communication domain (normative mean = 100, SD = 15), and receptive, expressive and written language subdomains (normative mean = 15, SD = 3) [49].

The Children's Communication Checklist Second Edition (CCC‐2) asked the parent to assess language in their child if the child spoke primarily in sentences [50]. The CCC‐2 is a parent assessment of 10 communication subdomains: speech, syntax, semantics, coherence, initiation, stereotyped, context, non‐verbal communication, social relations and interests (normative mean = 10, SD = 3). Scores of participants older than 16 years of age were generated using 16‐year‐old normative data.

The Communication Matrix described communication skills of participants who did not use spoken sentences to communicate [51]. The Communication Matrix portrays communication behaviours across four communicative functions (refuse, request, social, information) on a scale of 1 (pre‐intentional behaviour interpreted by the parent) to 7 (combining words, signs or symbols).

#### 2.3.3 Augmentative and Alternative Communication (AAC)

Parents provided information on augmentative and alternative communication (AAC, e.g. communication aids such as sign language and communication devices) use and perceptions of AAC.

#### 2.3.4 Clinician‐Reported Communication Outcome Scales

The CLN2 Clinical Rating Scale Language Domain (CLN2 CRS) clinician‐reported scale has scores 3 (‘apparently normal language. Intelligible and grossly age appropriate, no decline noted’), 2 (‘language has become recognizably abnormal: some intelligible words may form short sentences to convey concepts, requests or needs. This score signifies a decline from a previous level of ability [from the individual maximum reached by the child]’), 1 (‘hardly understandable. Few intelligible words’) and 0 (‘no intelligible words or vocalisations’) [52]. Higher scores indicate higher skills.

The Unified Batten Disease Rating Scale (UBDRS) is a clinician CLN3 disease scale with four domains: physical, behaviour, seizures and functional capability [25, 27]. The physical domain (total = 112 points) has 28 items, one of which is speech, each scored on a scale of 0 (normal) to 4 (severely impaired). Higher scores indicate lower skills. A speech pathologist assessed the CLN2 CRS language domain and UBDRS speech subdomain.

### 2.4 Adaptive Behaviour and Motor Skills

The Vineland‐3 also evaluated daily living skills, socialisation and motor skills. An adaptive behaviour composite (ABC) score was derived from communication, daily living skills and socialisation domains (normative mean = 100, SD = 15). Motor skill normative data was not available for individuals older than 9 years 11 months.

### 2.5 Statistical Analyses

Mann–Whitney *U* tests compared CLN2 and CLN3 disease scores on the Intelligibility in Context Scale. Wilcoxon Sign Rank Tests examined differences between receptive and expressive language skills. Confidence intervals and Mann–Whitney *U* tests compared acoustic speech measures in CLN3 disease and control participants. A Pearson's correlation coefficient assessed correlation between age and scores from the ChOMPS, PASSFP, Drooling Impact Scale, Intelligibility in Context, Vineland‐3, CCC‐2 and Communication Matrix. A Pearson's correlation coefficient assessed correlation between acoustic speech measures, age and average Intelligibility in Context Scores.

### 2.6 Ethical Approval

The study was approved by the Royal Children's Hospital Human Ethics Committee (HREC 37353A). Parents provided written, informed consent for individuals with Batten disease and child control participants. Adult control participants also provided written, informed consent. All parents who completed study information were the participants' biological mothers.

## 3 Results

### 3.1 Participants

Thirty‐three individuals with Batten disease participated from Australia (*n* = 15), United States (*n* = 13), Canada (*n* = 3), United Kingdom (*n* = 1) and Ireland (*n* = 1). There were six families with two affected children.

**FIGURE 1:**
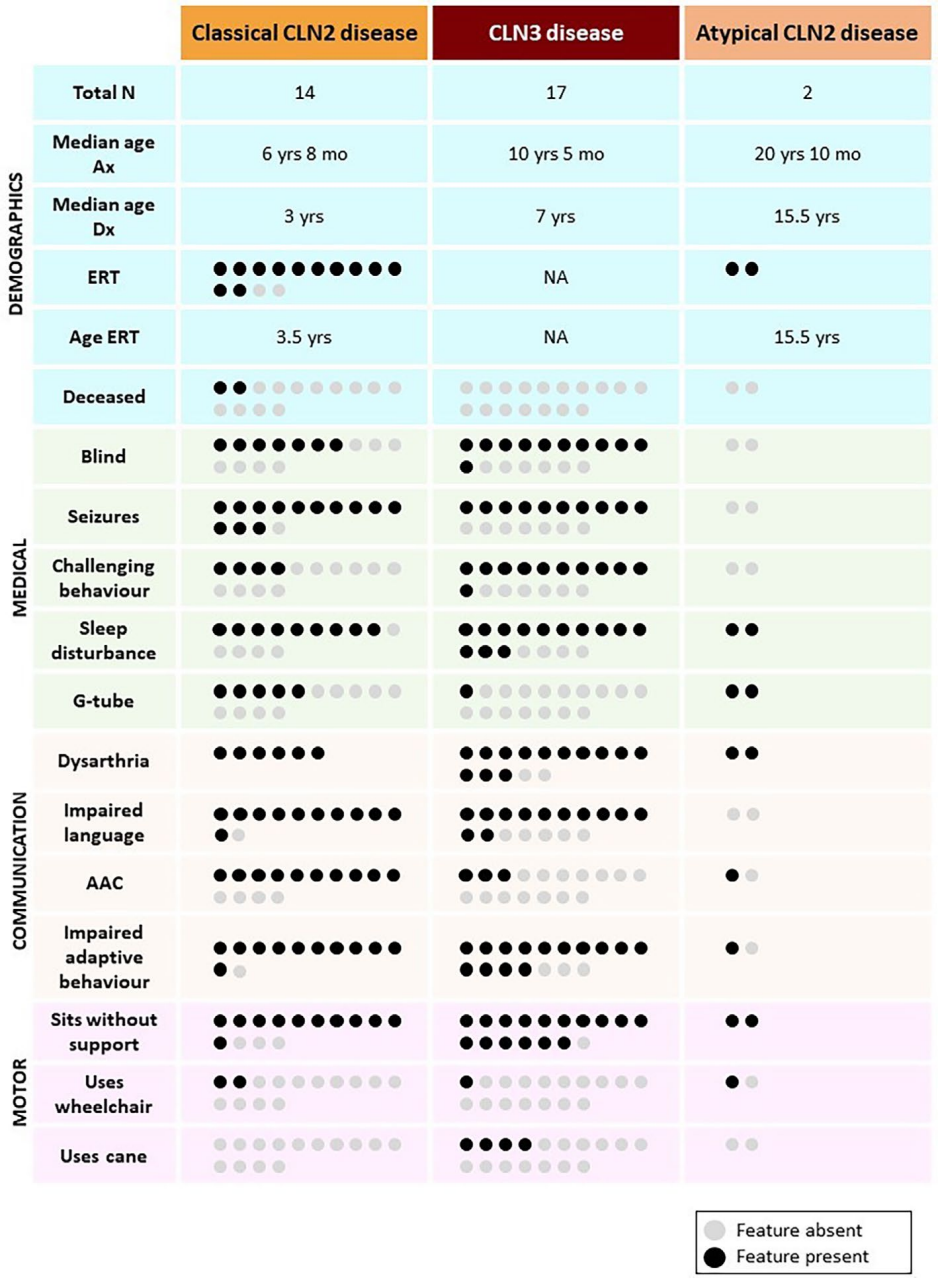
Phenotypes of participants with classical CLN2, CLN3 and atypical CLN2 disease. Circles represent individuals; grey circles = feature absent and black circles = feature present. AAC = augmentative and alternative communication system, Age ERT = average age starting ERT, Impaired adaptive behaviour = Vineland‐3 adaptive behaviour composite score < 85, Ax = assessed for this study, Dx = diagnosed, ERT = enzyme replacement therapy, Impaired language = Vineland‐3 communication domain score < 85, mo = months, N = number, NA = not applicable, yrs = years.

The median age of diagnosis was 3 years and 7 years for CLN2 and CLN3 disease, respectively. Sixteen participants (8 females) had CLN2 disease with a median age at study of 7.25 years (range 3–23 years); 17 participants (11 female) with CLN3 disease had a median age of 10 years 5 months (range 7–28 years) (Table 1, Figure 1). Two participants (P11, P14) with CLN2 disease passed away 2 years prior to the study, at 9 years of age.

**TABLE 1:** Characteristics and genotypes of participants with CLN2 and CLN3 disease.

Family ID	Participant ID	Age assessed (range, yrs)	Age diagnosed	Reason for genetic testing	Coding DNA	Protein	Length ERT (yrs)	CLN scale score
CLN2^a^								CLN2 CRS ML
FAM1	P1	10–11	18–23 mo	Older sibling diagnosed	c.509‐1G>C	—	7.8	1
					c.509‐1G>C			
FAM2	P2	4–5	3 yrs	Seizures, motor skills, stutter	c.509‐1G>C	—	1.4	1
					c.509‐1G>C			
FAM3	P3	4–5	4 yrs	Seizures	c.509‐1G>C	—	0.4	2
					c.509‐1G>C			
FAM4	P4	2–3	3 yrs	Seizures, language & global developmental delay	c.509‐1G>C	—	0.2	1
					c.509‐1G>C			
FAM5	P5	10–11	3 yrs	Seizures, speech/language delay	c.509‐1G>C	—	7.5	1
					c.509‐1G>C			
FAM6	P6	4–5	4 yrs	Seizures, speech/language delay, regression	c.622C>T	p.Arg208*	0.8	2
					c.622C>T	p.Arg208*		
FAM7	P7	4–5	3 yrs	Seizures	c.509‐1G>C	—	2	2
					c.622C>T	p.Arg208*		
FAM8	P8	6–7	4 yrs	Seizures	c.605C>T	p.Pro202Leu	2.9	0
					c.622C>T	p.Arg208*		
FAM9	P9	8.71	3 yrs	Seizures, fine motor & speech/language delay	c.851G>T	p.Gly284Val	4.7	0
					c.622C>T	p.Arg208*		
	P10	6–7	18–23 mo	Older sibling diagnosed	c.851G>T	p.Gly284Val	4.5	3
					c.622C>T	p.Arg208*		
FAM10	P11^c^	8–9	4 yrs	Seizures, motor skills	c.851G>T	p.Gly284Val	—	0
					c.622C>T	p.Arg208*		
FAM11	P12	6–7	4 yrs	Seizures, speech/language delay	c.472C>T	p.Gln158*	3.7	0
					c.622C>T	p.Arg208*		
	P13	2–3	7–11 mo	Older sibling diagnosed	c.472C>T	p.Gln158*	2.4	3
					c.622C>T	p.Arg208*		
FAM12	P14^c^	8–9	3 yrs	Seizures, ataxic movement, regression	c.1094G>A	p.Cys365Tyr	—	0
					c.1417G>A	p.Gly473Arg		
FAM13	P15	22–23	18 yrs	Ataxic movement, vision changes	c.508+4A>G	—	5.5	3
					c.837C>G	p.Tyr279*		
	P16	18–19	13 yrs	Older sibling diagnosed	c.508+4A>G	—	5.1	3
					c.837C>G	p.Tyr279*		
CLN3^b^								UBDRS Speech
FAM14	P17	8–9	5 yrs	Vision changes	c.461‐280_677+382del	—	—	1
					c.461‐280_677+382del			
FAM15	P18	6–7	7 yrs	Vision changes	c.461‐280_677+382del	—		1
					c.461‐280_677+382del			
FAM16	P19	6–7	6 yrs	Vision changes	c.461‐280_677+382del	—		3
					c.461‐280_677+382del			
FAM17	P20	12–13	5 yrs	Vision changes	c.461‐280_677+382del	—		3
					c.461‐280_677+382del			
FAM18	P21	10–11	7 yrs	Vision changes	c.461‐280_677+382del	—		2
					c.461‐280_677+382del			
	P22	8–9	7 yrs	Vision changes, older sibling diagnosed	c.461‐280_677+382del	—		0
					c.461‐280_677+382del			
FAM19	P23	12–13	7 yrs	Vision changes, regression	c.461‐280_677+382del	—		2
					c.461‐280_677+382del			
FAM20	P24	20–21	14 yrs	Seizures	c.461‐280_677+382del	—		4
					c.461‐280_677+382del			
	P25	10–11	7 yrs	Older sibling diagnosed	c.461‐280_677+382del	—		1
					c.461‐280_677+382del			
FAM21	P26	10–11	9 yrs	Vision changes	c.461‐280_677+382del	—		0
					c.461‐280_677+382del			
FAM22	P27	6–7	7 yrs	Vision changes	c.461‐280_677+382del	—		0
					c.461‐280_677+382del			
FAM23	P28	8–9	6 yrs	Vision changes	c.461‐280_677+382del	—		1
					c.461‐280_677+382del			
FAM24	P29	8–9	7 yrs	Vision changes	c.461‐280_677+382del	—		1
					c.461‐280_677+382del			
FAM25	P30	28–29	16 yrs	Movement disorder, regression	c.461‐280_677+382del	—		4
					c.566G>C	p.Gly189Ala		
FAM26	P31	16–17	12 yrs	Vision changes	c.461‐280_677+382del	—		0
					c.680A>G	p.Tyr227Cys		
	P32	12–13	8 yrs	Older sibling diagnosed	c.461‐280_677+382del	—		0
					c.680A>G	p.Tyr227Cys		
FAM27	P33	10–11	7 yrs	Vision changes	c.883G>A	p.Glu295Lys		1
					c.461‐280_677+382del	—		

Abbreviations: CLN2 CRS = CLN2 Clinical Rating Scale Language Domain score (3 = no decline noted, 0 = no intelligible words), ERT = enzyme replacement therapy, mo = months, UBDRS Speech = Unified Batten Disease Rating Scale Speech subdomain score (0 = normal, 4 = severely impaired), yrs = years, — = not applicable.

^a^ NM_000391.4.

^b^ NM_001042432.1.

^c^ Deceased < 2 years prior to study commencing, parents provided retrospective data of their most recent recollections of their child.

14/16 participants (88%) had classical CLN2 disease and two (12%) had atypical CLN2 disease, as determined by genotype and clinical presentation. Eleven participants with CLN2 disease (10/16, 63%) were compound heterozygotes, and six were homozygotes (6/16, 38%). Five homozygotes had the splice acceptor variant c.509‐1G>C. Eight participants (8/14, 57%) with classical CLN2 disease had the nonsense variant p.Arg208*. Two participants (FAM13) presented with an atypical, protracted form of CLN2 disease with residual TPP1 enzyme activity. Their variants were c.508+4A>G, predicted to lead to donor site loss after exon 5 (Splice AI: 0.69 score), and protein truncating variant p.Tyr279*, both predicted to be disease‐causing [53, 54].

Of the 34 CLN3 alleles in our 17 participants, 30 alleles were a recurrent 1 kb deletion, that was homozygous in 13/17 (76%) individuals. Four participants with CLN3 disease (4/17, 24%) were compound heterozygotes with the recurrent 1 kb deletion and a pathogenic missense variant. Missense variants were p.Gly189Ala and p.Glu295Lys in two individuals, and p.Tyr227Cys in siblings.

### 3.2 Medical

Common presentations leading to genetic testing for individuals with CLN2 disease included seizures (11/16, 69%), speech/language changes (7/16, 44%) and motor features (5/16, 31%) (Table 1). Four participants (4/16, 25%) were referred for testing following an older sibling's diagnosis. CLN3 participants were referred to genetic testing due to vision changes (13/17, 76%), skill regression (2/17, 12%) and an older sibling's diagnosis (3/17, 18%).

Fourteen participants with CLN2 disease (14/16, 88%) were receiving ERT (Table 1, Figure 1). P10, P13 and P16 (3/16, 19%) received pre‐symptomatic ERT as their older siblings had been diagnosed. Participants with classical CLN2 disease commenced ERT at a median of 3.5 years (range 9 months–4 years). For the 12 participants with classical CLN2 disease who had received ERT, the duration of ERT administration ranged from 7 years (P1, P5) to < 1 year (P3, P4, P6) (median = 3 years, 3 months, range 2 months–7 years, 9 months). The two deceased participants had not received ERT (2/16, 13%).

At assessment, 7/16 (44%) CLN2 and 11/17 (65%) CLN3 participants were legally blind (Figure 1, Table S3). 13/16 (81%) participants with CLN2 disease and 10/17 (59%) participants with CLN3 disease had seizures. Of those with CLN2 disease, only the two with atypical CLN2 disease (FAM13) and one with pre‐symptomatic ERT (P10) did not have seizures. 9/16 (56%) participants with CLN2 disease had cerebellar atrophy on magnetic resonance imaging (MRI) early in their disease course.

Challenging behaviours occurred in 4/16 (25%) participants with CLN2 disease and 11/17 (65%) participants with CLN3 disease (Figure 1). Participants with CLN2 disease had hyperactivity (3/16, 19%), aggression (2/16, 13%), impulsivity (2/16, 13%), anxiety (2/16, 13%), restricted interests and behaviour (1/16, 6%) and obsessions (1/16, 6%). Participants with CLN3 disease had anxiety (11/17, 65%), attentional difficulties (6/17, 35%), aggression (6/17, 35%), repetitive behaviour (5/17, 29%), obsessions (4/17, 24%), hyperactivity (3/17, 18%) and impulsivity (3/17, 18%).

Sleep disturbances were common in participants with CLN2 (11/16, 69%) and CLN3 (13/17, 76%) disease (Figure 1, Figure S1). The most frequent sleep disturbances in participants with CLN2 disease were frequent waking (7/16, 44%), followed by early waking and difficulty falling asleep (both 4/16, 25%). Conversely, participants with CLN3 disease had difficulty falling asleep (10/17, 59%) and frequent waking (8/17, 47%). Additional medical and milestone data are available in Tables S3 and S4.

#### 3.2.1 Feeding

8/15 (53%) participants with CLN2 disease ate solely orally and completed the ChOMPS; 7/15 (47%) participants were partially or completely enterally fed and completed the PASSFP (Figure S2, Table S3). In classical CLN2 disease, age was not associated with ChOMPS (*r* = −0.69, *p* = 0.06, median = 120/126, range 102–126) or PASSFP score (*n* = 5, *r* = 0.12, *p* = 0.84, median = 33/66, range 33–48) in this cohort. Five participants (5/14, 36%) with classical CLN2 disease had a G‐tube in situ. Participants with atypical CLN2 disease (FAM13) ate orally but also had G‐tubes in situ. They had high scores on the PASSFP indicating relatively low feeding difficulties (P15 = 61/66, P16 = 62/66).

Sixteen participants (16/17, 94%) with CLN3 disease completed the ChOMPS. Age was associated with ChOMPS score (*r* = −0.82, *p* = 0.0001, median = 124/126, range 40–126). P30 was the only individual with CLN3 disease who was enterally fed (1/17, 6%, G‐tube from 27 years old, PASSFP 52/66).

Of all the participants who completed the PASSFP (*n* = 8), only the two (25%) who had died were unable to feed orally prior to their death. For the 6/8 (75%) with enteral feeding who also fed orally, four coughed or gagged sometimes (4/6, 67%) but maintaining oral hygiene was relatively easy (6/6, 100%).

The Drooling Impact Scale recorded worsening with age (classical CLN2 *r* = 0.56, *p* = 0.04; CLN3 *r* = 0.86, *p* < 0.0001) (Table S3). Whilst some participants had severe drooling (CLN2 3/16, 19%; CLN3 2/17, 12%), they were largely unaware of it. For P9 and P11 (2/33, 6%), drooling severely impacted the child's and parent's everyday life.

Appetite disturbances (13/33, 39%) mainly comprised low appetite (CLN2 6/16, 38%; CLN3 3/17, 18%) or increased appetite (CLN2 1/16, 6%; CLN3 2/17, 12%).

### 3.3 Communication

8/13 (62%) participants with classical CLN2 disease had a language impairment prior to diagnosis (Table S4). P13's data is not considered here as she received a genetic diagnosis and ERT before age 12 months when first words are developed. A further 2/13 (15%) participants had plateauing in language development between 2 and 3 years. Six participants (6/17, 35%) with CLN3 disease had delayed speech and language milestones and an additional two participants (2/17, 12%) had speech disorders prior to diagnosis.

In CLN2 disease, parents reported language changes such as language regression (11/16, 69%), loss of vocabulary (11/16, 69%) and word‐finding difficulties (4/16, 25%, Table S4). Speech changes included declining intelligibility (4/16, 25%) and stuttering (3/16, 19%). Stuttering was more common in CLN3 disease (9/17, 53%). Parents of individuals with CLN3 disease noted early challenges with grammatical concepts such as past tense (4/17, 24%). Across both groups, 26/33 (79%) participants had seen a speech pathologist for communication support. Parents highlighted the importance of adapting their communication style to support their child's communication, including communication partner training skills of giving simple instructions and allowing extra processing time (13/33, 39%).

**FIGURE 2:**
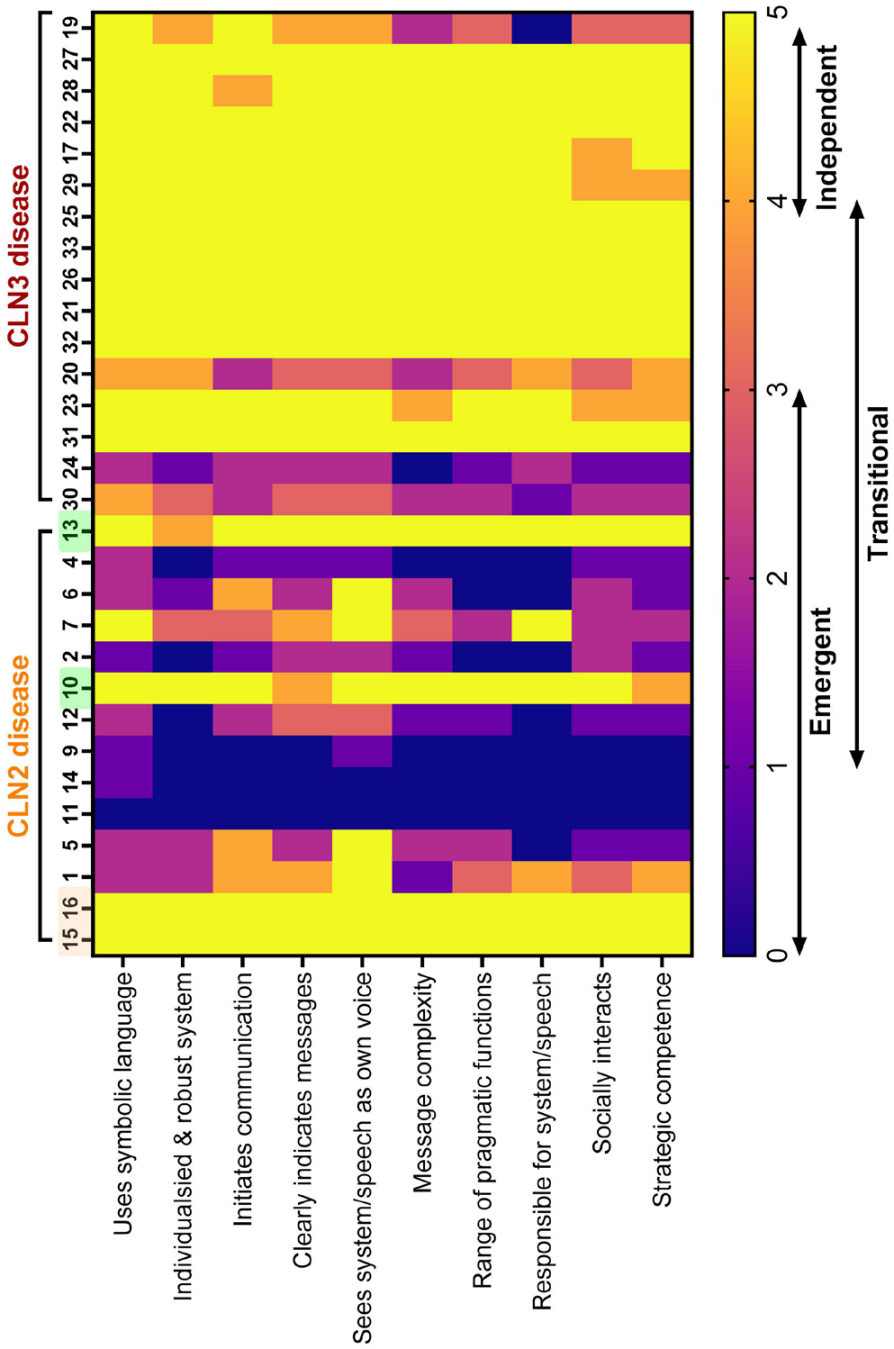
Roadmap of Communicative Competence. Roadmap of Communicative Competence (ROCC) scores across 10 communicative competencies in participants with CLN2 (*n* = 14) and CLN3 (*n* = 16) disease ordered from oldest to youngest (left to right). In CLN2 disease, two participants highlighted in yellow are atypical cases, and the two participants highlighted in green are classical cases who had ERT pre‐symptomatically. Emergent, transitional and independent communication defined by the ROCC; Emergent communicators typically use behaviours, gestures, facial expressions and body language and often require a familiar person to interpret their communication; Transitional communicators have some symbolic communication and communicate best with familiar people; Independent communicators can communicate autonomously with unfamiliar people.

Fourteen participants with CLN2 and 16 with CLN3 disease completed the ROCC (total *n* = 30, Figure 2). 14/30 (47%) participants used connected speech as their primary communication mode (CLN2 5/14, 36%; CLN3 9/16, 56%). In CLN2 disease, 2/5 (40%) with atypical CLN2 disease and 2/5 with pre‐symptomatic ERT (40%) used connected speech. Individuals with CLN3 disease had relative strengths in communicative competence compared to those with CLN2 disease.

#### 3.3.1 Speech

**FIGURE 3:**
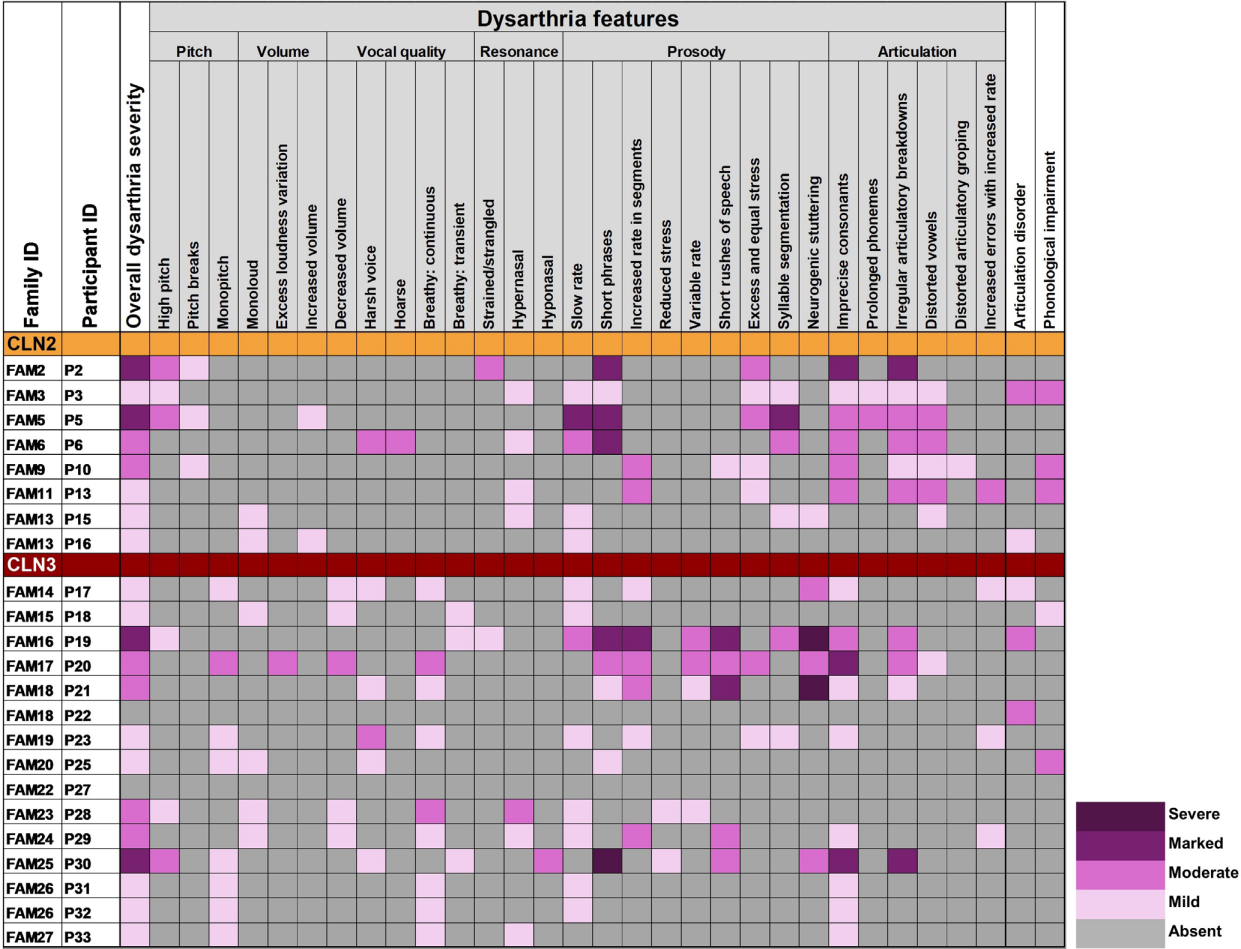
Perceptual speech features in participants with CLN2 (*n* = 8) and CLN3 disease (*n* = 15). Dysarthria symptoms assessed using the Mayo Clinic Dysarthria Rating System [45], and articulation and phonological impairments assessed using Dodd's [14] system for speech disorder differential diagnosis.

All verbal participants with CLN2 disease had dysarthria (8/8, 100%, Figures 1 and 3). One child with pre‐symptomatic ERT (P10) also had childhood apraxia of speech and a phonological disorder. P15 with atypical CLN2 disease had mild stuttering.

13/15 (87%) assessed participants with CLN3 disease had dysarthria (Figures 1 and 3, Table 2), including moderate to severe neurogenic stuttering in 5/15 (33%). Stuttering was primarily at the beginning of phrases and included sound, part‐word, word and phrase repetitions, with all occurring in 2/15 (13%). 2/15 (13%) participants with CLN3 disease had a phonological delay and 3/15 (20%) an articulation disorder. FAM26 had subclinical dysarthric features. In CLN2 and CLN3 disease, the most common dysarthric features were imprecise consonants (16/23, 70%), slow rate (13/23, 57%) and short phrases (9/23, 39%).

**TABLE 2:** Acoustic speech analysis in participants with CLN3 disease and control participants.

Stimulus	Measure^b^	CLN3 disease		Controls^c^		Mann–Whitney *U* test	*P*‐value^d^
		Mean	SD	Mean	SD		
Counting^a^	Pause mean	0.20	0.11	0.16	0.08	517	0.10
	Syllable duration mean	0.28	0.12	0.23	0.07	436	0.08
	Articulation rate	4.10	3.90	3.83	0.94	426	0.06
Alternating motion rate DDK	Voice onset time mean	0.03	0.01	0.03	0.01	441	0.32
	Articulation rate	6.35	6.13	6.37	2.68	412	0.05
	Syllable duration mean	0.19	0.08	0.16	0.06	346	0.006**
Sequential motion rate DDK	Voice onset time mean	0.04	0.01	0.04	0.01	404	0.08
	Articulation rate	4.53	1.36	5.20	1.53	381	0.04**
	Syllable duration mean	0.21	0.05	0.18	0.06	370	0.03**
Sustained vowel	Fundamental frequency mean	276.20	50.55	245.00	61.75	362	0.03**
	Fundamental frequency SD	50.15	33.00	21.72	22.48	236	< 0.0001**
	MFCC1 mean	377.20	106.3	365.30	177.60	471	0.39
	MFCC2 mean	−108.5	109.0	−80.83	113.30	456	0.30

Abbreviations: DDK = diadochokinetic, MFCC = Mel‐frequency cepstral coefficient, SD = standard deviation.

^a^ Counting 1–10.

^b^ Recorded and analysed using Redenab Pty Ltd. software.

^c^ Two age‐ and sex‐matched controls per participant with CLN3 disease.

^d^ Mann–Whitney *U* test.

** Statistically significant *p* < 0.05.

13/17 with CLN3 disease completed the acoustic speech battery (Table S2, Data S1). Participants with CLN3 disease were different from same sex and age‐matched controls on five measures (Table 2); they had longer syllable duration and slower articulation rate on diadochokinetic tasks and higher and more variable fundamental frequency on a sustained vowel task, reflecting poor vocal control. Acoustic measures reflected perceptual features of imprecise and slow speech, alongside high pitch and pitch breaks. In participants with CLN3 disease, there was no correlation with age (*p* > 0.05), in contrast with the control group where syllable duration and articulation rate increased whilst vowel fundamental frequency decreased with age (*p* < 0.05) (Data S1). Diadochokinetic tasks examining syllable duration and articulation rate (“pataka”) distinguished CLN3 participants from controls more than automatic speech tasks, for example, counting. A small sample size (3/16) of participants with CLN2 disease precluded statistical analysis (Data S1).

Participants with classical CLN2 disease had lower intelligibility (median = 3 ‘sometimes’ understood, range 1–4), than those with CLN3 disease (median = 4 ‘usually’ understood, range 2–5) (*U* = 51.5, *p* = 0.006, Table 1). Age did not correlate with intelligibility for participants with classical CLN2 disease (*r* = −0.39, *p* = 0.17), but did for participants with CLN3 disease (*r* = −0.67, *p* = 0.004). Participants were understood better by their parents (classical CLN2 median = 4, range 1–5; CLN3 median = 5, range 2–5) than strangers (classical CLN2 median = 2 ‘rarely’, range 1–4; CLN3 median = 4, range 1–5). P16 with atypical CLN2 disease and pre‐symptomatic ERT, was ‘always’ understood. 7/17 (41%) participants with CLN3 disease were always understood.

#### 3.3.2 Language

**FIGURE 4:**
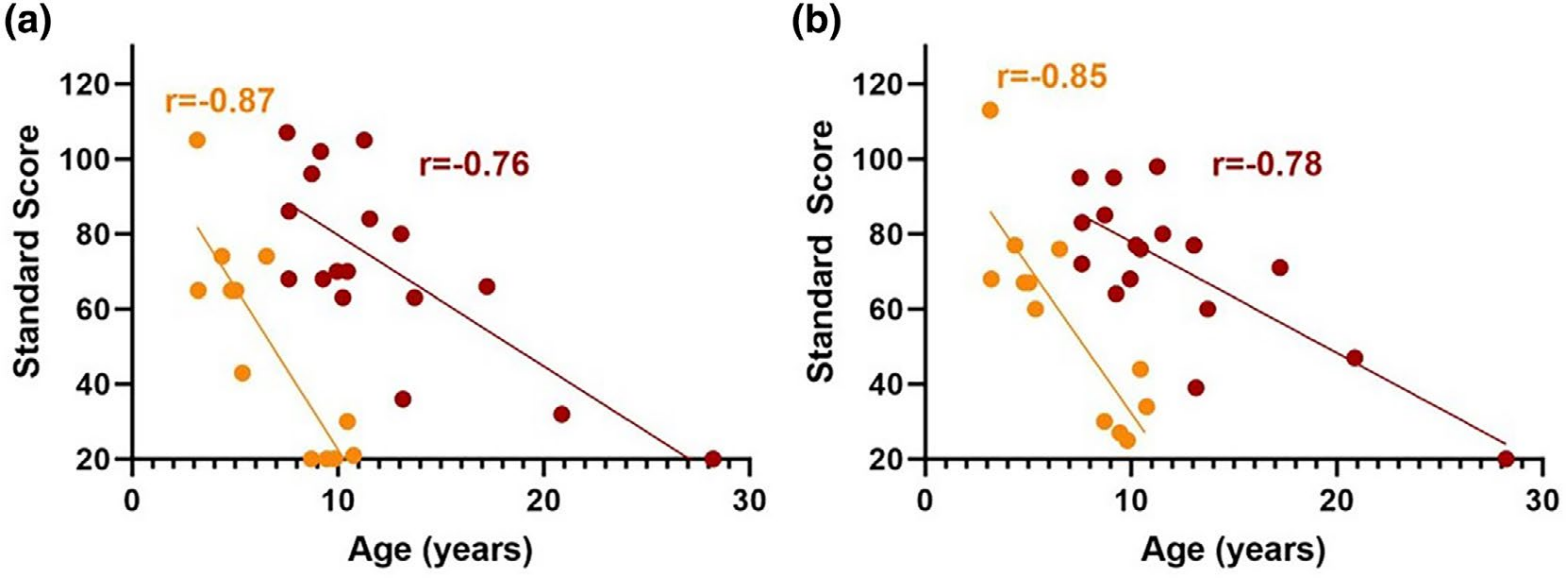
(a) Vineland‐3 score communication domain and age in participants with classical CLN2 disease and CLN3 disease. Communication domain scores on the Vineland Adaptive Behaviour Scales 3rd Edition (Vineland‐3) (normative mean = 100, normative SD = 15). Participants with classical CLN2 disease (yellow): *n* = 12, *r* = −0.87, 95% CI = −0.96 to −0.60, *R* ^2^ = 0.77, *p* = 0.0002. Participants with CLN3 disease (red): *n* = 17, *r* = −0.76, 95% CI = −0.91 to −0.43, *R* ^2^ = 0.57, *p* = 0.0005. (b) Vineland‐3 score adaptive behaviour composite and age in classical CLN2 disease and CLN3 disease. Adaptive Behaviour Composite scores on the Vineland Adaptive Behaviour Scales 3rd Edition (Vineland‐3) (normative mean = 100, normative SD = 15). Participants with classical CLN2 disease (yellow): *n* = 12, *r* = −0.85, 95% CI = −0.96 to −0.55, *R* ^2^ = 0.73, *p* = 0.0004. Participants with CLN3 disease (red): *n* = 17, *r* = −0.78, 95% CI = −0.92 to −0.47, *R* ^2^ = 0.60, *p* = 0.0002.

Thirty‐one participants completed the Vineland‐3 (CLN2 *n* = 14; CLN3 *n* = 17) (Figures 1 and 4). Communication scores were impaired in participants with CLN2 (mean = 57, SD = 31) and CLN3 (mean = 72, SD = 25) disease, whereas 3/14 (21%) with atypical CLN2 disease or pre‐symptomatic with ERT (P13), had average communication skills. 5/17 (29%) participants (P18, P22, P26–28) with CLN3 disease also had average communication skills. Participants with CLN2 disease did not have a difference between their receptive (mean = 6, SD = 5) and expressive language skills (mean = 6, SD = 5, *Z* = 0, *p* > 0.99). Conversely, participants with CLN3 disease had stronger expressive language (mean = 12, SD = 4) than receptive (mean = 9, SD = 6, *Z* = 2.77, *p* = 0.004). Vineland‐3 communication skills declined with age in both diseases (Figure 4).

**FIGURE 5:**
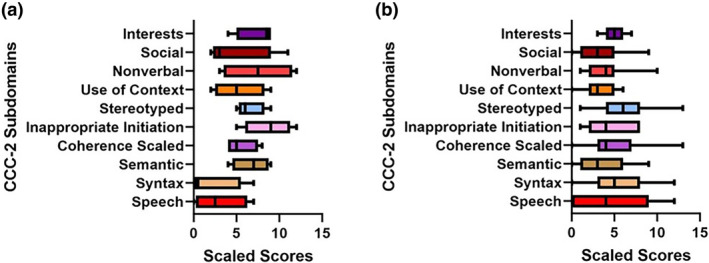
(a) CCC‐2 subdomains in participants with classical CLN2 disease. Children's Communication Checklist 2nd Edition (CCC‐2) scores in participants with classical CLN2 disease (*n* = 4) subdomain scores (normative mean = 10, SD = 3): Speech (mean = 3, SD = 3.17), Syntax (mean = 2, SD = 3.37), Semantic (mean = 6.80, SD = 2.22), Coherence (mean = 5.50, SD = 1.92), Inappropriate initiation (mea*n* = 8.75, SD = 2.87), Stereotyped (mean = 6.50, SD = 1.73), Use of context (mean = 5.25, SD = 2.99), Non‐verbal (mean = 7.50, SD = 4.20), Social (mea*n* = 4.75, SD = 4.19) and Interests (mean = 7.50, SD = 2.38). Differences between subdomains: *χ* ^2^ = 21.66, *p* = 0.01. Middle line = median, bottom whisker = minimum, upper whisker = maximum. (b) CCC‐2 subdomains in participants with CLN3 disease. Children's Communication Checklist 2nd Edition (CCC‐2) scores in participants with CLN3 disease (*n* = 15) subdomain scores (normative mean = 10, SD = 3): Speech (mean = 4.93, SD = 4.18), Syntax (mean = 5.27, SD = 3.83), Semantic (mean = 3.53, SD = 3.07), Coherence (mean = 4.93, SD = 3.13), Inappropriate initiation (mean = 4.67, SD = 2.53), Stereotyped (mean = 6.20, SD = 3.41), Use of context (mean = 3.27, SD = 2.15), Non‐verbal (mean = 4.13, SD = 2.39), Social (mean = 3.27, SD = 2.82) and Interests (mean = 5.07, SD = 1.16). Differences between subdomains: *χ* ^2^ = 28.86, *p* = 0.0007. Middle line = median, bottom whisker = minimum, upper whisker = maximum.

4/14 (29%) participants with classical and two with atypical CLN2 disease completed the CCC‐2 (Figure 5). Semantic skills (*r* = −0.98, *p* = 0.02), pragmatic skills of initiation (*r* = −0.98, *p* = 0.02) and use of context (*r* = −0.98, *p* = 0.02) declined with age. The CCC‐2 also reflected declining pragmatic skills with the use of context (*r* = −0.69, *p* = 0.005) and interests (*r* = −0.72, *p* = 0.002) in 15 participants with CLN3 disease.

**FIGURE 6:**
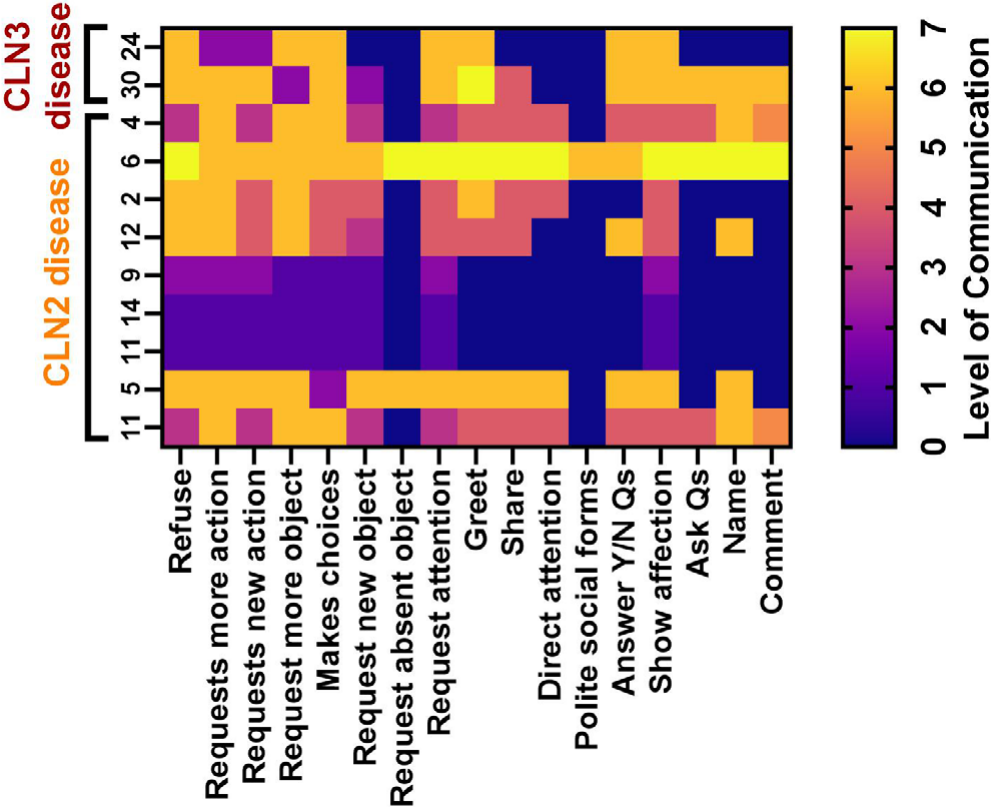
Communication Matrix scores. Communication Matrix scores across communicative functions in participants with classical CLN2 (*n* = 9) and CLN3 (*n* = 2) disease who do not use connected speech to communicate ordered from oldest to youngest (left to right). Level 0 = does not display, Level 1 = pre‐intentional behaviour, Level 2 = intentional behaviour, Level 3 = unconventional communication, Level 4 = conventional communication, Level 5 = concrete symbols, Level 6 = abstract symbols (e.g. single words, single signs) and Level 7 = language (e.g. combining words or symbols). Qs = questions, Y/N = Yes/No. Classical CLN2 disease differences between communicative functions mastered: *χ* ^2^ = 19.64, *p* = 0.0006.

9/14 (64%) participants with classical CLN2 disease completed the Communication Matrix (Figure 6), which revealed comparative strengths in requesting ‘more’. All participants showed affection, but most could not communicate information or for social reasons. They had relative strengths in refusing but found communicating information challenging. The Communication Matrix skills mastered did not decline with age (*r* = −0.34, *p* = 0.36). 2/17 (12%) participants with CLN3 disease could refuse but had difficulties requesting (Figure 6).

#### 3.3.3 Augmentative and Alternative Communication (AAC)

11/16 (69%) participants with CLN2 disease and 3/17 (18%) with CLN3 disease had AAC (Table S5). Four participants (4/33, 12%) had a dedicated AAC system, accessed by direct finger point (2/33, 6%) or partner‐assisted scanning (2/33, 6%). However, no participants used AAC as their primary communication mode. Only 8/14 (57%) parents felt that their child's speech pathologist had adequate AAC knowledge to support their child, and 6/33 (18%) parents thought that AAC actually hindered speech.

#### 3.3.4 Clinician‐Reported Communication Outcome Scales

CLN2 CRS scores were impaired (median = 1, range 0–3, Table 1). Only two participants with classical CLN2, who had commenced ERT pre‐symptomatically, and two atypical cases, showed no decline on the CLN2 CRS. Scores declined with age in classical CLN2 disease (*r* = −0.60, *p* = 0.02).

Only the two individuals with CLN3 disease older than 20 years had severely impaired communication skills (2/17, 12%). FAM26 had relatively unimpaired communication on their UBDRS scores for their age. UBDRS scores (median = 1, range 0–4) also showed declining communication skills with age (*r* = 0.58, *p* = 0.01).

### 3.4 Adaptive Behaviour and Motor Skills

Vineland‐3 ABC scores declined with age in both diseases (Figure 4, classical CLN2 *n* = 12, *r* = −0.85, *p* = 0.0004; CLN3 *n* = 17, *r* = −0.78, *p* = 0.0002). Motor skills declined with age in classical CLN2 disease (*n* = 10, *r* = −0.90, *p* = 0.0004) but not in CLN3 disease (*n* = 7, *r* = −0.62, *p* = 0.14). Several participants with CLN2 and CLN3 disease had visual loss, compounding impairments on fine motor (e.g. drawing) and gross motor (e.g. running) tasks. P13 who commenced ERT pre‐symptomatically had average motor skills.

In terms of motor skills, 13/16 (81%) participants with CLN2 disease and 16/17 (94%) with CLN3 disease could sit unaided. Some participants had a relatively typical gait (CLN2 5/16, 31%; CLN3 6/17, 35%). Others had an abnormal or unstable gait (CLN2 2/16, 13%; CLN3 3/17, 18%), walked short distances (CLN2 2/16, 13%; CLN3 2/17, 12%), walked short distances but primarily used a wheelchair (CLN2 3/16, 19%; CLN3 1/17, 6%) or no longer walked (CLN2 4/16, 25%; CLN3 1/17, 6%). 4/17 participants (24%) with CLN3 disease used a cane.

## 4 Discussion

We have systematically characterised speech, language and feeding in the two most common types of Batten disease, CLN2 and CLN3, identifying different speech and language profiles between the two conditions. Speech and language impairments prior to diagnosis were common in participants with CLN2 disease and observed in almost half of those with CLN3 disease. All individuals with CLN2 disease had dysarthria, and older individuals with classical CLN2 disease became non‐verbal. Individuals who commenced ERT pre‐symptomatically and those with atypical CLN2 disease had stronger speech and language skills. In participants with classical CLN2 disease, there was no difference between receptive and expressive language skills. In participants with CLN3 disease, expressive language skills were stronger than receptive language, and dysarthria frequently manifested as neurogenic stuttering. In CLN3 disease, cognitive ability and attention may have contributed to receptive language impairment [24, 55]. Speech, cognition, language and adaptive behaviour skills declined with age in both diseases. Lower Vineland‐3 scores in CLN2 disease reflect a more rapid disease course than CLN3 disease, even with ERT in CLN2 disease.

Children with childhood dementias often present with speech and language impairments at diagnosis, similar to our cohort with Batten disease [13, 56]. Whilst speech and language disorders are well established presenting features in CLN2 disease, they are also important in CLN3 disease [13]. Previous CLN2 disease cohorts have reported higher incidence of speech and language impairments before genetic diagnosis, suggesting underdiagnosis of early speech and language impairments in our cohort (Table S1). Prior to genetic diagnosis, speech and language impairments were also noted in 47% of individuals with CLN3 disease, building on the communication difficulties previously reported prior to genetic diagnosis in three individuals with CLN3 disease [28]. Increased awareness of speech and language presentations in Batten disease may reduce the length of the diagnostic odyssey for families. For example, they may present with typical acquisition of first words followed by slowed or incomplete attainment of combining words, onset of neurogenic stuttering or speech and language regression. Early diagnosis is also increasingly critical with the advent of disease‐modifying treatments like ERT [57]. Clinicians who are often first consulted for children's speech and language disorders, such as speech pathologists, general practitioners and paediatricians, should be aware of the possibility of childhood dementia. Longitudinal systematic assessment could elucidate the developmental trajectory of speech and language features to alert clinicians to the possibility of a childhood dementia [14, 20, 58, 59].

Neurogenic stuttering in our cohort was characteristic of dysarthria rather than isolated, developmental stuttering [60, 61]. Unlike developmental stuttering, neurogenic stuttering develops suddenly and is caused by neurologic damage. In addition, stuttering behaviours are primarily sound and syllable repetitions unaffected by concurrent task execution (e.g. stuttering less when reading); secondary stuttering behaviours and sound blocks are rare [60, 61]. In CLN3 disease, neurogenic stuttering was a core manifestation of dysarthria, with onset in the first decade of life, considerably earlier than fine and gross motor impairments develop [62, 63, 64]. In addition, one participant with atypical CLN2 disease also had neurogenic stuttering, consistent with the dysarthria reported in atypical CLN2 disease [65]. Historically, in children with Batten disease speech manifestations have been regarded as an unrelated developmental speech impairment, rather than being recognised as a specific disease feature. Misdiagnosis of developmental stuttering may result in ineffective speech therapy designed to treat the wrong disorder. Comprehensive speech assessment is therefore essential to ensure that children receive appropriately targeted therapy.

AAC is regarded as a key tool to enable communication in individuals with minimal or unclear speech [66]. Whilst some individuals had access to AAC, none used it as their primary communication method. This may be due to several reasons. Firstly, some caregivers believed that AAC hindered their child speaking and also had concerns that their speech pathologist had inadequate knowledge to prescribe appropriate AAC. Secondly, disentangling fine motor skills, visual impairment and linguistic and cognitive decline is challenging in Batten disease and many AAC systems have limited capacity to adapt to neurodegenerative conditions involving visual and motor loss [67, 68]. Thirdly, learning AAC may prove challenging in view of the high carer burden for parents [69, 70, 71]. AAC should be delivered proactively in the context of the whole child, and consider the complex, often competing needs of individuals with Batten disease and their families.

Like AAC, communication partner training should be offered early in an individual's disease course. Parents highlighted the benefits of adapting their communication style to support their child's expressive and receptive language skills. Given the complex nature of Batten disease, it is essential to facilitate caregivers' communication with their child. Communication partner training is also useful to support challenging behaviours, frequently observed in individuals with CLN3 disease [28, 55, 72]. Challenging behaviours often eclipse physical disease symptoms as the most difficult aspect of care for families, often in association with declining communication and cognition [26, 55, 73, 74]. In adult‐onset dementia, communication partner training has been associated with reduced challenging behaviours and improved wellbeing [75]. A holistic approach is recommended, including early access to communication partner training and AAC, that can adapt to an individual's changing needs to maintain speech and language skills for as long as possible.

Despite an often‐homogenous genotype in individuals with CLN3 disease, we observed marked speech and language heterogeneity. For instance, P19 and P26 were similar ages and shared the same homozygous 1 kb deletions; P19 had severe dysarthria and language impairment, whilst P27 had average language skills without dysarthria. Heterogenous speech and language features in individuals with CLN3 disease, despite uniform genotype, reflect previously reported phenotypic variation in this population and other monogenic disorders [8, 26, 38]. We also describe a novel *CLN3* variant c.566G>C and the associated phenotype.

In classical CLN2 disease, initiation of pre‐symptomatic ERT has only been reported in two children [76, 77]. We contribute a further two individuals who received pre‐symptomatic ERT, including the youngest child to date who was 9 months old when ERT was initiated and at 3 years of age had a speech disorder without language impairment. The two previously reported cases both showed improving language skills although had mild language impairment. Here we have documented the first individual to commence pre‐symptomatic ERT with average language skills, highlighting the potential importance of early therapeutic intervention for optimal communication outcomes. We also report the first case with atypical CLN2 disease to commence ERT pre‐symptomatically. Further phenotyping is required to examine the influence of pre‐symptomatic ERT on long term speech and language outcomes.

Clinician‐reported communication outcome measures (CLN2 CRS and UBDRS) currently fail to ascertain subtle speech and language changes in individuals with CLN2 and CLN3 disease. These measures failed to capture speech or language impairment in the two children with classical CLN2 disease who had commenced ERT pre‐symptomatically, yet both individuals had speech disorders, and one had a language impairment. Similarly, a child with CLN3 disease may have a stable score on a clinician‐reported communication outcome measure, despite the changing nature and severity of their dysarthria and language impairment. Using these measures alone may also not successfully capture speech and language changes in atypical CLN2 disease, as they have a relatively protracted disease course compared to classical CLN2 disease. These scales also do not differentiate between speech, expressive language and non‐verbal communication (e.g. AAC), nor do they capture receptive language skills. Clinician‐reported communication outcome measures may falsely suggest absence or stability of speech and language skills, particularly in individuals with classical CLN2 disease and pre‐symptomatic commencement of ERT, atypical CLN2 disease and CLN3 disease. Conversely, comprehensive bi‐annual speech and language measures would equitably assess speech and language progression and treatment responses in all individuals with CLN2 or CLN3 disease.

Age has been historically used as a proxy for disease progression, however, this approach needs to be applied judiciously. For example, older individuals with CLN2 disease did not always exhibit more severe disease, particularly those with atypical presentations, and individuals with classical CLN2 disease who had ERT.

Some individuals had speech but could not complete the acoustic speech battery tasks due to cognitive, linguistic and behavioural challenges. Assessments with high task demand were virtually impossible in individuals with advanced disease [5]. Conversely, conversation samples had relatively minimal task demand. Future studies should focus on low‐demand tasks for objective acoustic and linguistic analysis, such as spontaneous speech and vocalisations, alongside standardised caregiver report measures [78, 79, 80]. Assessments should also consider limitations of questions that rely on vision (e.g. questions about understanding gesture). Assessment in longitudinal studies could also elucidate further details of speech and language features across the disease course. To better capture comprehensive longitudinal data, future research could assess speech and language in larger CLN2 and CLN3 cohorts, including non‐English‐speaking participants from high prevalence locations such as Scandinavia and Europe. Additional investigation of the relationship between speech and language progression, non‐verbal cognition and seizure activity could further explain neurodegeneration in these populations.

In conclusion, we elucidate the speech, language and feeding phenotypes of individuals with CLN2 and CLN3 disease using a battery of clinician and caregiver‐administered assessments. Speech and language skills reflect disease onset and progression, as well as changes in response to treatment. Our findings underscore the importance of speech and language interventions in individuals with Batten disease, including tailored therapy to support speech and language skills for as long as possible, early access to adaptable AAC and communication partner training.

## Author Contributions

Conceptualization: Lottie D. Morison, Ineka T. Whiteman, Lisa Tilbrook, Ingrid E. Scheffer and Angela T. Morgan. Data curation: Lottie D. Morison, Adam P. Vogel, Joanna Bredebusch, Ruth Braden and Michael S. Hildebrand. Formal analysis: Lottie D. Morison, Adam P. Vogel and Michael S. Hildebrand. Funding acquisition: Lottie D. Morison, Ineka T. Whiteman, Lisa Tilbrook, Michael C. Fahey, Ingrid E. Scheffer, Angela T. Morgan and Adam P. Vogel. Investigation: Lottie D. Morison. Methodology: Lottie D. Morison, Ineka T. Whiteman, Adam P. Vogel, Lisa Tilbrook, Michael C. Fahey, Ingrid E. Scheffer and Angela T. Morgan. Project administration: Lottie D. Morison and Angela T. Morgan. Resources: Angela T. Morgan and Adam P. Vogel. Software: Adam P. Vogel and Angela T. Morgan. Supervision: Ingrid E. Scheffer and Angela T. Morgan. Writing – original draft: Lottie D. Morison, Ingrid E. Scheffer and Angela T. Morgan. Visualisation: Lottie D. Morison. Writing – review and editing: all authors.

## Ethics Statement

The study was approved by the Royal Children's Hospital Human Ethics Committee (HREC 37353A).

## Consent

Parents provided written, informed consent for participants with Batten disease. Adult control participants and parents of child control participants provided written informed consent.

## Conflicts of Interest

Lisa Tilbrook, Michael C. Fahey, Ingrid E. Scheffer and Angela T. Morgan are on the Batten Disease Support and Research Association Australia Medical and Scientific Advisory Board. Angela T. Morganis the director of paediatric/neurodevelopment communication at Redenlab Pty Ltd. and Adam P. Vogel is the Chief Science Officer and Founder of Redenlab Pty Ltd. Ineka T. Whiteman is Head of Research and Medical Affairs, Batten Disease Support, Research and Advocacy Foundation; Principal Scientific Consultant, Beyond Batten Disease Foundation; Head of Research and Medical Affairs, Batten Disease Support and Research Association Australia. Ingrid E. Scheffer is a consultant for Biohaven Pharmaceuticals, Care Beyond Diagnosis, Cerecin Inc., Eisai, Epilepsy Consortium, Longboard Pharmaceuticals, UCB and Zynerba Pharmaceuticals. Ingrid E. Scheffer has received payment honoraria and travel support from Biocodex, BioMarin, Chiesi, Eisai, GlaxoSmithKline, Liva Nova, Nutricia, Stoke Therapeutics, UCB and Zuellig Pharma. Ingrid E. Scheffer is on scientific advisory boards for Bellberry Ltd., BioMarin, Chiesi, Eisai, Encoded Therapeutics, Garvan Institute of Medical Research, Knopp Biosciences, Longboard Pharmaceuticals, UCB and Takeda Pharmaceuticals. Ingrid E. Scheffer is a director at Bellberry Ltd., Australian Academy of Health and Medical Sciences and Australian Council of Learned Academies Ltd. Ingrid E. Scheffer is a trial investigator for Anavex Life Sciences, Cerebral Therapeutics, Cerecin Inc., Cereval Therapeutics, Eisai, Encoded Therapeutics, EpiMinder Inc., ES‐Therapeutics, GW Pharmaceuticals, Marinus Pharmaceuticals, Neuren Pharmaceuticals, Neurocrine BioSciences, Ovid Therapeutics, Takeda Pharmaceuticals, UCB, Ultragenyx, Xenon Pharmaceuticals, Zogenix and Zynerba Pharmaceuticals. Ingrid E. Scheffer has a patent issued for # Molecular diagnostic/theranostic target for benign familial infantile epilepsy (BFIE) [PRRT2] 2011904493 & 2012900190 and PCT/AU2012/001321 (TECH ID:2012–009) *# SCN1A* testing held by Bionomics Inc. and a patent pending for WO61/010176 (filed: 2008): Therapeutic Compound. The other authors declare no conflicts of interest.

## Supporting information


Figure S1.

Figure S2.

**Data S1.** Supporting Information.

Table S1.

Table S2.

Table S3.

Table S4.

Table S5.

## Data Availability

The data that support the findings of this study are available on request from the corresponding author. The data are not publicly available due to privacy or ethical restrictions.
