# Supplementary material for: Speech, Language and Non‐verbal Communication in CLN2 and CLN3 Batten Disease
Source: J Inherit Metab Dis. 2025 Jan 16;48(1):e12838. doi: 10.1002/jimd.12838 (PMC11739554; doi:10.1002/jimd.12838)
Supplement: Supplementary file 1 — Figure S1. [file JIMD-48-0-s007.pdf]

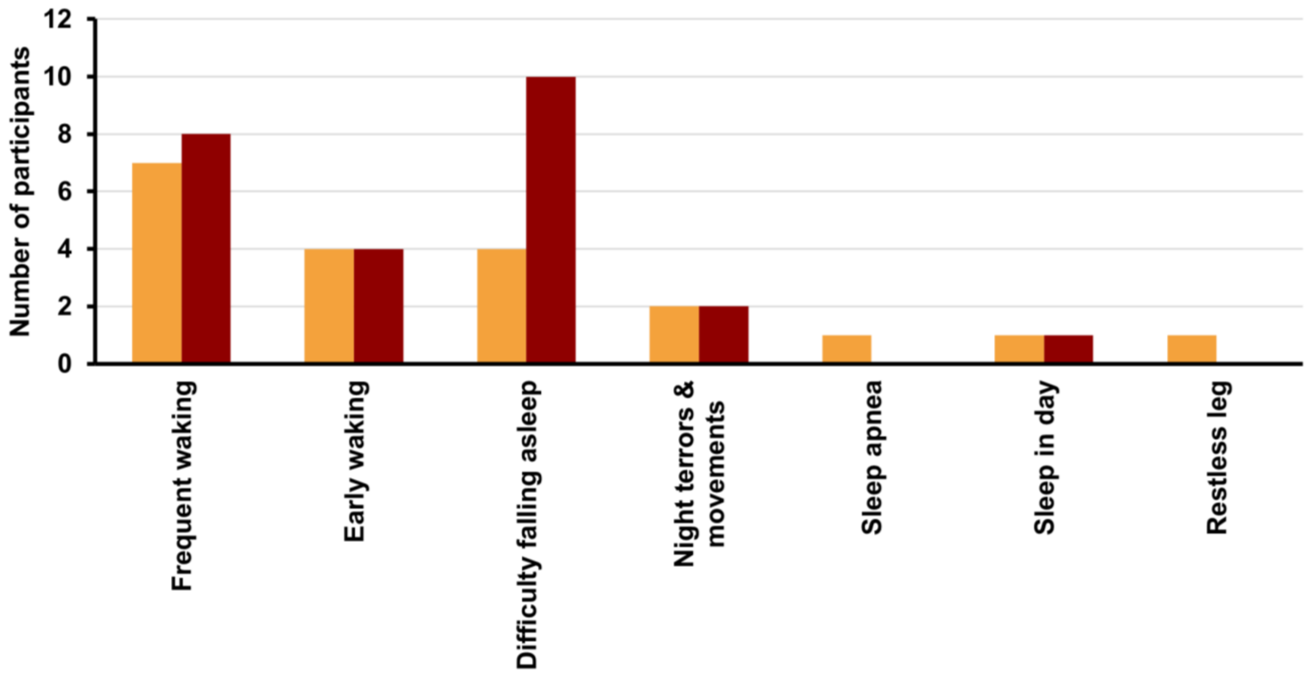

Supplemental Figure 1. Types of sleep disturbance in participants with CLN2 and CLN3 disease

Prevalence of sleep disturbance in participants with CLN2 (yellow, 11/16, 69%) and CLN3 (red, 13/17, 76%) disease, and types of disturbance as reported by parents.
