# Supplementary material for: Speech, Language and Non‐verbal Communication in CLN2 and CLN3 Batten Disease
Source: J Inherit Metab Dis. 2025 Jan 16;48(1):e12838. doi: 10.1002/jimd.12838 (PMC11739554; doi:10.1002/jimd.12838)
Supplement: Supplementary file 2 — Figure S2. [file JIMD-48-0-s008.pdf]

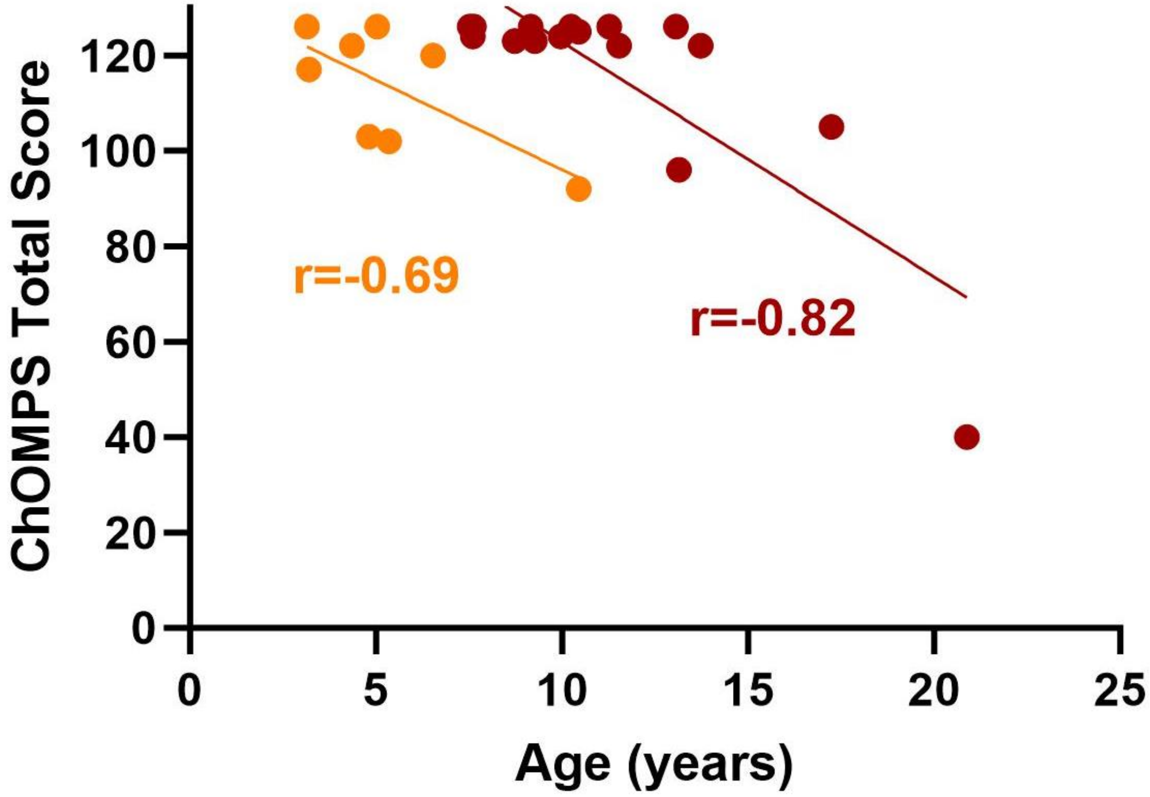

Supplemental Figure 2. ChOMPS scores and age in participants with classical CLN2 and CLN3 disease

Age and total scores on the Child Oral Motor Proficiency Scale (ChOMPS) in participants with classical CLN2 (yellow, n=8, r=-0.69, p=0.06) and CLN3 (red, n=16, r=-0.82, p=0.0001) disease.
