# Supplementary material for: Speech, Language and Non‐verbal Communication in CLN2 and CLN3 Batten Disease
Source: J Inherit Metab Dis. 2025 Jan 16;48(1):e12838. doi: 10.1002/jimd.12838 (PMC11739554; doi:10.1002/jimd.12838)
Supplement: Supplementary file 3 — Data S1. Supporting Information. [file JIMD-48-0-s006.pdf]

Supplemental Results. Acoustic analysis of speech in participants with CLN2 and CLN3 disease

Group demographics

Three participants (n=3) with CLN2 disease (median age=18 years, Q1-Q3=10.5–20 years) completed the acoustic analysis speech battery. Two English speaking age and sex matched controls per participant (n=6, median age=18.5 years, Q1-Q3=9 years 8 months – 19.5 years) also completed the acoustic analysis speech battery. There was no different between the ages of the groups (U=7, p=0.71, Figure 1). Two participants with atypical CLN2 disease, and one participant received pre-symptomatic enzyme replacement therapy.

Thirteen participants (n=13) with CLN3 disease (median=10 years, 5 months, Q1-Q3=8 years, 2 months – 12.25 years) completed the acoustic analysis speech battery. Two English speaking age and sex matched controls per participant (n=26, median=11.25 years, Q1-Q3=8 years, 10 months to 13 years, 4 months) also completed the acoustic analysis speech battery. There was no different between the ages of the groups (U=132, p=0.28, Figure 2).

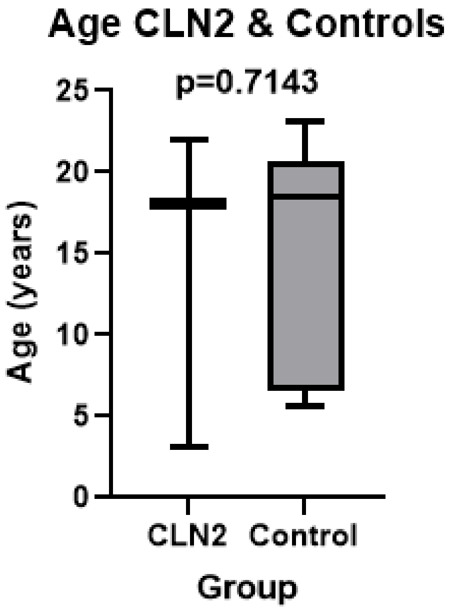

Figure 1. Ages of participants with CLN2 disease (n=3) and control group (n=6) with acoustic data, U=7, p=0.71.

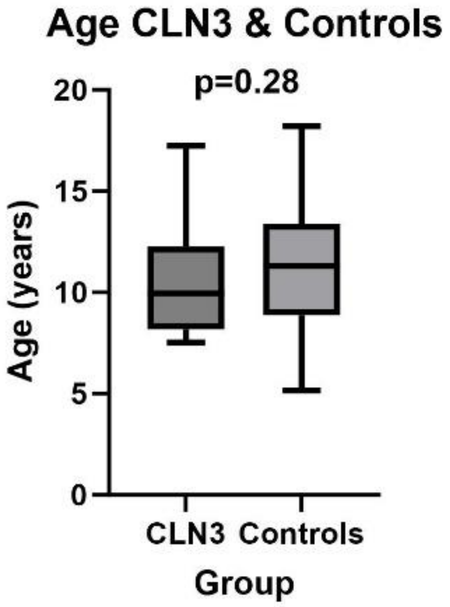

Figure 2. Ages of participants with CLN3 disease (n=13) and control group (n=26) with acoustic data, U=132, p=0.28.

Acoustic analysis results

Small sample size of participants with CLN2 disease who completed the acoustic speech battery precluded statistical analysis (n=3, FAM13 and P13). Consequently, descriptive statistics of participants with CLN2 disease's acoustic speech measures are outlined in Table 1. P13 did not complete the counting or sequential motion rate diadochokinetic tasks. Participants with CLN2 disease had a longer syllable duration and slower articulation rate on both diadochokinetic tasks than controls. However, this was not reflected on the counting task.

Participants with CLN3 disease (n=13) were statistically significantly different from controls (n=26) on four measures ($p<0.05$); average syllable duration on alternating and sequential motion rate diadochokinetic rate tasks, average articulation rate on sequential motion rate task, and average and standard deviation of fundamental frequency on a sustained vowel task. Like in CLN2 disease, participants with CLN3 disease's syllable duration and articulation rate did not differ greatly from control participants on the counting task. Participants with CLN3 disease had longer syllable duration and slower articulation rate than control participants on diadochokinetic tasks, alongside a higher and more variable fundamental frequency. These measures were compared to average scores on the Intelligibility in Context Scale to

identify potential correlations. Average syllable duration on diadochokinetic rate tasks and average Intelligibility in Context Scale scores were weakly correlated (alternating motion rate: r=0.08, 95% CI=-0.49 to 0.61, R^2=0.007, p= 0.79; sequential motion rate: r=0.07, 95% CI=-0.53 to 0.62, R^2=0.004, p= 0.84). Likewise, correlations between sequential motion rate average articulation rate and average Intelligibility in Context Scale scores were weak (r=0.35, 95% CI=-0.28 to 0.77, R^2=0.12, p=0.27). Individual average and standard deviation fundamental frequency on a prolonged vowel task also had a weak correlation with intelligibility (average: r=0.35, 95% CI=-0.28 to 0.77, R^2=0.12, p=0.27, individual standard deviation: r=-0.29, 95% CI=-0.74 to 0.34, R^2=0.08, p=0.36). In CLN3 disease, age did not significantly correlate with any of the five measures that were statistically different from controls (Figure 3). Conversely, for control participants, age correlated with three of these five measures (Figure 4).

Table 1. Acoustic speech analysis of participants with CLN2 disease and controls

Stimulus	Measure†	CLN2 disease		Controls‡		CLN2 disease > Controls
		Mean	SD	Mean	SD	
Counting*	Pause mean	0.19	0.09	0.23	0.11	N
	Syllable duration mean	0.27	0.06	0.30	0.10	N
	Articulation rate	3.33	0.18	3.18	0.59	Y
Alternating motion rate DDK	Articulation rate	4.98	1.24	6.26	2.11	N
	Syllable duration mean	0.15	0.08	0.13	0.03	Y
Sequential motion rate DDK	Articulation rate	4.17	0.51	5.57	2.03	N
	Syllable duration mean	0.22	0.07	0.16	0.07	Y
Sustained vowel	Fundamental frequency mean	201.20	75.74	200.1	95.95	Y
	Fundamental frequency SD	21.36	21.84	12.05	16.35	Y
	MFCC1 mean	480.00	130.30	333.60	115.70	Y
	MFCC2 mean	-176.80	172.10	-46.80	86.55	Y

*Counting 1-10, †Recorded and analysed using Redenab Pty Ltd software, ‡Two age and sex matched controls per participant with CLN3 disease, §Mann-Whitney U Test,

**=statistically significant p<0.05, DDK=Diadochokinetic, MFCC=Mel-frequency cepstral coefficient, SD=standard deviation. P13 did not completed counting 1-10 and sequential motion rate tasks.

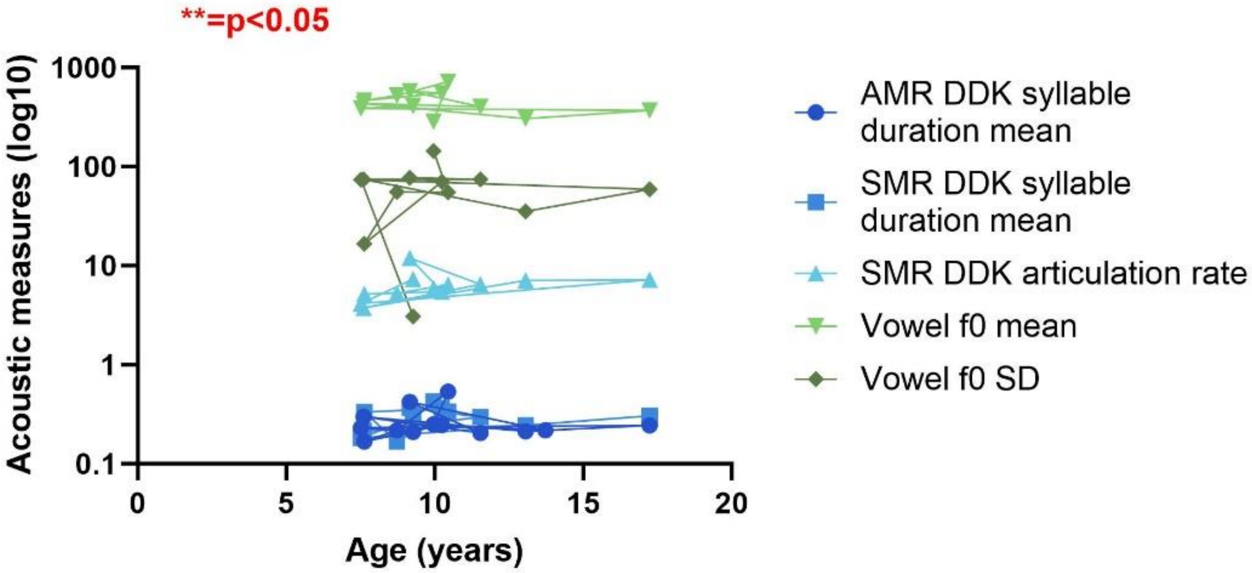

Figure 3. Acoustic measures and age in participants with CLN3 disease. Correlation between age and acoustic measures (log10) in participants with CLN3 disease (n=13) **=p<0.05 AMR DDK syllable mean: (r=-0.07, 95% CI=-0.60 to 0.50, R^2=0.01, p=0.81). SMR syllable duration mean: (r=0.18, 95% CI=-0.44 to 0.68, R^2=0.03, p=0.58). SMR DDK articulation rate: (r=0.29, 95% CI=-0.34 to 0.74, R^2=0.09, p=0.35). Vowel f0 mean: (r=-0.26, 95% CI=-0.72 to 0.37, R^2=0.07, p=0.42). Vowel f0 SD: (r=-0.00, 95% CI=-0.57 to 0.57, R^2=0.00, p=0.99)

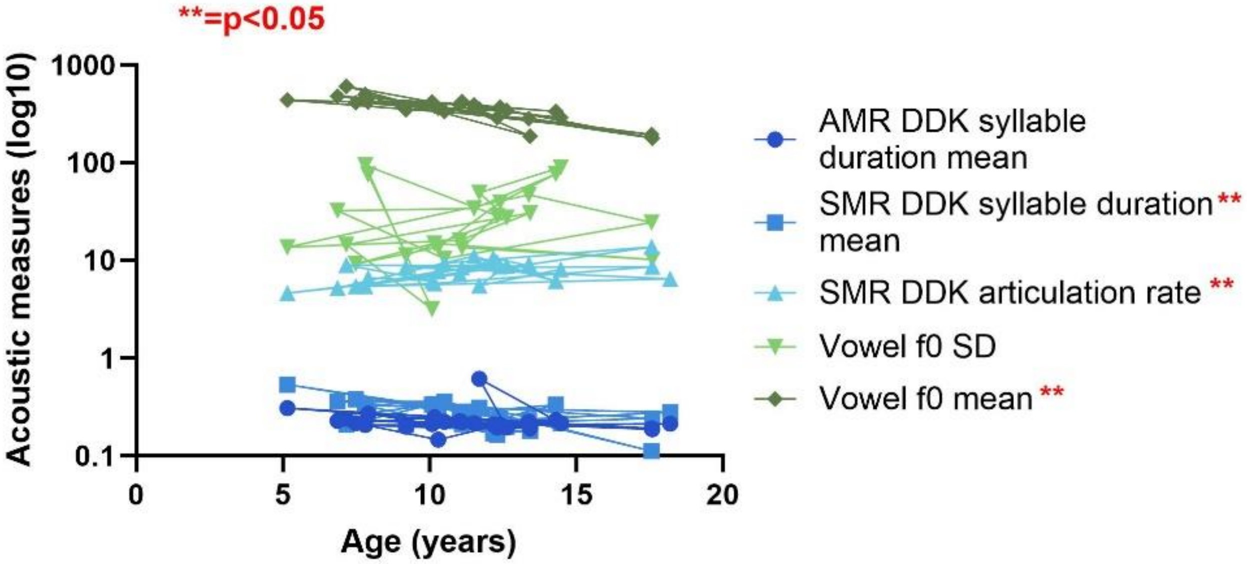

Figure 4. Acoustic measures and age in CLN3 age and sex matched control participants. Correlation between age and acoustic measures (log10) in age and sex matched control participants (n=26). AMR DDK syllable mean: (r=-0.16, 95% CI=-0.51 to 0.25, R^2=0.02, p=0.45). SMR syllable duration mean: (r-0.57=, 95% CI=-0.78 to -0.24, R^2=0.33, p=0.002). SMR DDK articulation rate: (r=0.50, 95% CI=0.14 to 0.74, R^2=0.25, p=0.01). Vowel f0 mean: (r=-0.84, 95% CI=-0.92 to -0.66, R^2=0.71, p=<0.0001). Vowel f0 SD: (r=0.11, 95% CI=-0.31 to 0.49, R^2=0.01, p=0.61)

AMR DDK syllable mean: alternating motion rate diadochokinetic task syllable duration mean. SMR syllable duration mean: sequential motion rate diadochokinetic task syllable duration mean. SMR DDK articulation rate: sequential motion rate diadochokinetic task articulation rate. Vowel f0 SD: sustained vowel fundamental frequency standard deviation. Vowel f0 mean: sustained vowel fundamental frequency mean.
