# Supplementary material for: Speech, Language and Non‐verbal Communication in CLN2 and CLN3 Batten Disease
Source: J Inherit Metab Dis. 2025 Jan 16;48(1):e12838. doi: 10.1002/jimd.12838 (PMC11739554; doi:10.1002/jimd.12838)
Supplement: Supplementary file 4 — Table S1. [file JIMD-48-0-s002.pdf]

## Supplemental Table 1. Speech and language delay as an initial symptom in CLN2 disease

| Proportion of individuals with speech/language features at disease onset | Description of speech/language features | Language/speech most common first symptom | Reference |
|---|---|---|---|
| Classical phenotype: 18/20 (90%) | Language regression | Y | (Guelbert et al., 2024) |
| Atypical phenotype: 14/16 (87%) | Language delay | | |
| First words: 2/21 (10%) at >18 months or not achieved<br>Two-words phrases: 20/21 (95%) at >24 months or not achieved<br>Whole sentences: 23/23 (100%) at >36 months or not achieved | Description of milestones | NA | (Nickel et al., 2023) |
| 20/23 (87%) | Not achieving milestone of forming sentences | | |
| 18/22 (82%) | Language delay | Y | (Kravljanac & Tadic, 2022) |
| 15/18 (83%) | Language difficulties as first symptom (delay or regression) | Y | (Ho et al., 2022) |
| 18/30 (60%) | Speech delay before disease onset | N | (Ardicli et al., 2021) |
| 8/30 (27%) | Speech regression as main feature of disease onset | | |
| 7/8 (88%) | Language delay | Y | (Estublier et al., 2021) |
| 1/30 (3%) | Language delay | Y | (Lourenço et al., 2021)<br>*Atypical CLN2 disease* |
| 1/30 (3%) | Language regression | | |
| 2/30 (7%) | Language difficulties | | |
| 2/30 (7%) | Speech delay | | |
| 6/30 (20%) | Speech/language difficulties as an additional presenting symptom | | |
| 6/14 (43%) | Language abnormalities | Y | |

| | | | |
|---|---|---|---|
| 3/14 (21%) | Ataxia/language abnormalities | | (Wibbeler et al., 2021)<br>*Atypical CLN2 disease* |
| 10/13 (77%) | Language delay | Y | (Johnson et al., 2020) |
| 2/11 (18%) | Delay of language | N | (Dozières-Puyravel et al., 2020) |
| 4/5 (80%) | Speech delay | Y | (Biswas et al., 2020) |
| 1/12 (8%) | Language delay | N | (Aydin et al., 2020) |
| Language delay: 30/36 (83%).<br>First words: 6/36 (17%) at >18 months<br>Two-word phrases: 7/36 (19%) at >24 months<br>Whole sentences: 19/36 (53%) at >36 months | Early language delay and description of milestones | N | (Nickel et al., 2018) |
| Group 1: 4/12 (34%)<br>Group 2: 6/23 (26%) | Language delay | N | (Beltrán et al., 2018) |
| 5/12 (42%) | Speech delay | N | (Pérez-Poyato et al., 2013) |

N=No, Y=Yes
