# Supplementary material for: Speech, Language and Non‐verbal Communication in CLN2 and CLN3 Batten Disease
Source: J Inherit Metab Dis. 2025 Jan 16;48(1):e12838. doi: 10.1002/jimd.12838 (PMC11739554; doi:10.1002/jimd.12838)
Supplement: Supplementary file 5 — Table S2. [file JIMD-48-0-s005.pdf]

Supplemental Table 2. Acoustic speech battery

Task Name	Speech sub-systems acoustically analysed	Measures
Counting 1 – 10<br>(2 attempts)	Prosody	Pauses<br>Syllable duration<br>Articulation rate
Alternating motion rate “papapa”<br>(2 attempts)	Articulation<br>Prosody	Voice onset time<br>Syllable duration<br>Articulation rate
Sequential motion rate “pataka”<br>(2 attempts)	Articulation<br>Prosody	Voice onset time<br>Syllable duration<br>Articulation rate
Long Vowel /ɐː/<br>(2 attempts)	Phonation	Fundamental frequency<br>Mel-frequency cepstral coefficient 1<br>Mel-frequency cepstral coefficient 2

Note: these speech tasks, alongside conversation and single word tasks, did not require visual stimuli.
