# Supplementary material for: Speech, Language and Non‐verbal Communication in CLN2 and CLN3 Batten Disease
Source: J Inherit Metab Dis. 2025 Jan 16;48(1):e12838. doi: 10.1002/jimd.12838 (PMC11739554; doi:10.1002/jimd.12838)
Supplement: Supplementary file 6 — Table S3. [file JIMD-48-0-s001.pdf]

Supplemental Table 3. Health and medical data of participants with CLN2 and CLN3 disease

Family ID	Participant ID	Sex	Hearing impairment	Vision	Average DIS score	Sit without support	Walking	Tremor	Sleep disturbance	Neurodevelopmental diagnoses	Behavioural concerns	Seizures	Digestive problems	G-tube	Heart problems	Skeletal	Other health	Poor thermal regulation	SP	OT	PT
**CLN2**																					
**1**	**1**	M	-	Blind	3.1	+	Walked previously, no longer walks	+	+	-	-	+	Constipation	+	-	Inward turned feet		-	+	+	+
**2**	**2**	M	-	-	1.3	+	Walk for a limited period, or a limited distance	+	+	SPD	+	+	-	-	-	-		+	+	+	+
**3**	**3**	F	-	-	1	+	Walks typically	-	-	-	-	+	-	-	-	-		-	-	+	-
**4**	**4**	M	-	-	2.6	+	Walks typically	-	+	-	+	+	-	-	-	-		-	+	+	-
**5**	**5**	M	-	Blind	1.1	+	Uses a wheelchair but can	-	+	-	-	+	Constipation	-	-	Scoliosis		+	+	+	+

							walk short distances														
**6**	**6**	F	-	Blind	1.1	+	Walks with an abnormal gait	-	-	-	-	+	-	-	-	-		-	+	+	+
**7**	**7**	F	-	-	2	+	Walks typically	-	-	ADHD	+	+	Constipation	-	Murmur	-		-	+	+	-
**8**	**8**	M	-	-	3.3	+	Uses a wheelchair but can walk short distances	-	+	-	-	+	-	-	-	-		-	+	+	+
**9**	**9**	F	-	Blind	5.2	-	Walked previously, no longer walks	+	-	-	-	+	-	+	-	Scoliosis		-	+	+	+
**9**	**10**	M	-	Squint, Issues with depth perception	1	+	Walks typically	-	+	-	+	-	-	-	-	-		-	+	+	+
**10**	**11**	M	-	Blind	6.1	-	Walked previously, no longer walks	+	+	-	-	+	Constipation, vomiting	+	-	Scoliosis, kyphosis		+	+	+	+
**11**	**12**	F	-	Blind	3.3	+	Walks with an abnormal gait	+	+	-	-	+	Constipation,	+	-	Scoliosis	Acute liver failure	+	+	+	+

**11**	**13**	F	-	-	1.2	+	Walks typically	-	-	-	-	+	-	-	-	-		-	+	-	+
**12**	**14**	F	-	Blind	4	-	Walked previously, no longer walks	+	+	-	-	+	Constipation	+	-	Scoliosis		+	+	-	-
**13**	**15**	M	-	-	1	+	Uses a wheelchair but can walk short distances	+	+	ASD, SPD, Developmental coordination disorder	-	-	Constipation, pain	+	Abnormal EKG	Scoliosis		+	+	+	+
**13**	**16**	F	-	-	1	+	Walk for a limited period, or a limited distance	+	+	-	-	-	-	+	-	-	Menorrhagia, neutropenia	+	-	+	+
**CLN3**																					
**14**	**17**	M	-	Blind	1	+	Walk with cane	-	+	ASD	+	+	-	-	-	-		-	+	+	+
**15**	**18**	M	-	Blind	1	+	Walks typically	-	-	ASD	+	-	-	-	-	-		-	-	-	-
**16**	**19**	F	-	Blind	1	+	Walks with an abnormal gait	-	+	DCD	+	+	-	-	-	-	Premature Adenarche	+	+	-	-
**17**	**20**	M	-	Blind	1.1	+	Unstable walk	-	+	-	+	+	-	-	-	-		-	+	-	-

**18**	**21**	F	-	Blind	1	+	Walk with cane	-	+	ADHD	-	-	Constipation	-	-	-		-	+	+	+
**18**	**22**	F	-	Blind	1	+	Learning to walk with cane	-	-	-	-	-	-	-	-	-		-	+	+	+
**19**	**23**	F	-	Blind	1	+	Walk for a limited period, or a limited distance	+	+	SPD	+	+	Constipation	-	-	Scoliosis		-	+	+	+
**20**	**24**	F	-	Blind	3.6	-	Walked previously, no longer walks	+	+	-	-	+	-	-	-	-		-	+	+	-
**20**	**25**	F	-	Blind	1	+	Walks typically	-	+	ADHD	+	+	-	-	-	-		-	+	+	-
**21**	**26**	M	-	Nystagmus	1	+	Walk with cane	-	+	ADHD	-	+	-	-	-	-		+	+	+	+
**22**	**27**	F	-	Hypermetropia, myopia, strabismus, low vision	1	+	Walks typically	-	-	ADHD	+	-	-	-	-	-		-	+	+	-
**23**	**28**	M	-	Blind	1	+	Walks typically	-	+	-	+	-	Constipation	-	-	-		-	-	+	-
**24**	**29**	F	-	Hypermetropia, myopia,	2.3	+	Walks with an	-	+	-	+	-	-	-	-	-		-	-	+	-

				retinal degeneration			abnormal gait														
**25**	**30**	F	-	Blind	4.4	+	Uses a wheelchair but can walk short distances	+	+	-	-	+	Neurogenic bowel and cladder	+	-	Osteoporosis		+	+	+	+
**26**	**31**	F	-	Macular dystrophy	1.6	+	Walk for a limited period, or a limited distance	+	+	SPD	+	+	-	-	-	-		+	-	+	+
**26**	**32**	M	-	Macular dystrophy	1	+	Walks typically	-	+	-	+	-	-	-	-	-		+	-	-	+
**27**	**33**	F	Dx 10 yrs mild bilateral sensorineural hearing loss	Myopia, Macular dystrophy	1	+	Walks typically	-	-	ADHD	-	+	-	-	-	-		-	+	+	-

-=absent, +=present

ADHD=Attention Deficit Hyperactive Disorder, ASD=Autism Spectrum Disorder, C section=Caesarean Section, CVI=Cortical Visual Impairment, DCD=Developmental Coordination Disorder, DIS=Drooling Impact Scale (1, not at all – 10, always), Dx=Diagnosed, F=Female, G-tube=Gastrostomy Tube, M=Male, Kg=Kilograms, OT=Occupational Therapy, PT=Physiotherapy, SP=Speech Pathology, SPD=Sensory Processing Disorder, Wks=weeks
