# Supplementary material for: Speech, Language and Non‐verbal Communication in CLN2 and CLN3 Batten Disease
Source: J Inherit Metab Dis. 2025 Jan 16;48(1):e12838. doi: 10.1002/jimd.12838 (PMC11739554; doi:10.1002/jimd.12838)
Supplement: Supplementary file 7 — Table S4. [file JIMD-48-0-s004.pdf]

Supplemental Table 4. Speech and language milestones in participants with CLN2 and CLN3 disease

Family ID	Participant ID	Milestones*		Parent observed communication loss	Parent descriptions: Before diagnosis*		Parent descriptions: After diagnosis	
		First words (mo)	First sentences (yrs)		Language changes	Speech changes	Language changes	Speech changes
**CLN2**								
**FAM1**	**P1**	<12	NYA	Y	Language delay	-	Language regression, loss of vocabulary	-
**FAM2**	**P2**	<12	2-3	Y	Language delay, word finding difficulties, paraphasias	Stuttering	Language regression, loss of vocabulary, some improvement in response to SP therapy and ERT	
**FAM3**	**P3**	<12	2-3	N	-	-	-	-
**FAM4**	**P4**	NYA	NYA	N	Language delay	-	-	-
**FAM5**	**P5**	<12	NYA	Y	Language delay	-	Language regression, loss of vocabulary	Intelligibility declining
**FAM6**	**P6**	<12	2-3	Y	-	-	Language regression, loss of vocabulary, word finding difficulties	-
**FAM7**	**P7**	12-15	2-3	Y	Loss of vocabulary	Intelligibility declining	Language regression, loss of vocabulary, word finding difficulties, paraphasias	Intelligibility declining
**FAM8**	**P8**	12-15	2-3	Y	Loss of vocabulary	-	Language regression, loss of vocabulary	-
**FAM9**	**P9**	<12	2-3	Y	Language delay, selective mutism diagnosis	-	Language regression, loss of vocabulary	-
**FAM9**	**P10**	<12	2-3	N	-	-	Gaining language skills	-
**FAM10**	**P11**	<12	NYA	Y	Language delay 2yrs	Intelligibility affected	Language regression, loss of vocabulary	Intelligibility declining
**FAM11**	**P12**	<12	2-3	Y	Plateau at 3yrs, language regression and	-	Language regression, loss of vocabulary, word finding difficulties	Stutter at 3.5yrs

					word finding difficulties 4yrs			
**FAM11**	**P13**	<12	2-3	N	NA	NA	Gaining language skills, language improves after ERT	Stutter, treated with SP therapy
**FAM12**	**P14**	<12	2-3	Y	Plateau at 2yrs, language regression	-	Language regression, loss of vocabulary, word finding difficulties	Stutter, intelligibility declining
**FAM13**	**P15**	<12	2-3	Y	-	-	Language regression, loss of vocabulary	Pronunciatio n difficulties
**FAM13**	**P16**	<12	2-3	Y	-	-	Longer processing in conversation	Slower speech
**CLN3**								
**FAM14**	**P17**	15-18	2-3	Y	-	-	-	Stuttering
**FAM15**	**P18**	<12	2-3	N	-	-	-	-
**FAM16**	**P19**	12-15	2-3	Y	Language delay	Stuttering at 3.5yrs	Slowed language skill development	Worsening dysfluency
**FAM17**	**P20**	<12	2-3	Y	-	Some pronunciation difficulties	Language regression	Intelligibility declining, stuttering
**FAM18**	**P21**	<12	2-3	Y	-	-	Slowed language skill development	Stuttering, mumbling
**FAM18**	**P22**	12-15	2-3	N	-	-	-	-
**FAM19**	**P23**	≥18	2-3	Y	Language delay	Speech disorder	Language regression, word finding difficulties, paraphasias, grammatical difficulties	Intelligibility declining, stuttering
**FAM20**	**P24**	<12	2-3	Y	-	-	Language regression	Intelligibility declining
**FAM20**	**P25**	<12	2-3	N	-	-	-	-
**FAM21**	**P26**	12-15	2-3	Y	-	-	Grammatical difficulties	Stuttering
**FAM22**	**P27**	<12	4-5	Y	-	-	Gaining language skills	-
**FAM23**	**P28**	<12	2-3	Y	-	-	Language regression, loss of vocabulary, word finding difficulties	Stuttering, mumbling

**FAM24**	**P29**	<12	2-3	Y	-	-	Language regression, loss of vocabulary, grammatical difficulties	Stuttering, slurring
**FAM25**	**P30**	<12	2-3	Y	-	Monotone speech	Perseveration, word finding difficulties, paraphasias	Intelligibility declining
**FAM26**	**P31**	<12	2-3	Y	-	-	Loss of vocabulary, grammatical difficulties, word finding difficulties, paraphasias	Stuttering
**FAM26**	**P32**	<12	2-3	N	-	-	-	-
**FAM27**	**P33**	<12	2-3	N	-	-	-	-

*=language delay classified as 1st words achieved >12 months old, combining words >4-5 years old and/or if parents reported language delay, -=Not reported by parent, ERT=Enzyme replacement therapy, Mo=Months, N=No, NYA=Not yet achieved, SP=Speech Pathology therapy, Y=Yes, Yrs=Years
