# Supplementary material for: Speech, Language and Non‐verbal Communication in CLN2 and CLN3 Batten Disease
Source: J Inherit Metab Dis. 2025 Jan 16;48(1):e12838. doi: 10.1002/jimd.12838 (PMC11739554; doi:10.1002/jimd.12838)
Supplement: Supplementary file 8 — Table S5. [file JIMD-48-0-s003.pdf]

Supplemental Table 5. AAC use in participants with CLN2 and CLN3 disease

**Family ID**	**Participant ID**	**Aided AAC (present or previous)**		**Unaided AAC (present or previous)**		**Present primary communication mode**	**Communication strategies parents found useful**
		**Aided AAC**	**Access method**	**Natural gesture***	**Single sign**		
**CLN2**							
**FAM1**	**P1**	N	-	Y	Y	Speech (limited words - less than 20)	On body sign
**FAM2**	**P2**	Y	Single pictures	Y	Y	Body language and/or bodily movements and/or facial expressions, and/or vocalisations	Key word sign, gesture, pointing, pictures/symbols
**FAM3**	**P3**	N	-	N	N	-	-
**FAM4**	**P4**	N	-	Y	N	Body language and/or bodily movements and/or facial expressions, and/or vocalisations	-
**FAM5**	**P5**	Y	Partner assisted scanning	N	N	Speech (more than 20 words)	-
**FAM6**	**P6**	N	-	Y	N	Speech (more than 20 words)	Giving yes/no options
**FAM7**	**P7**	N	-	Y	N	Speech (connected speech - uses sentences)	Communication partner adaptations e.g., speaking slowly
**FAM8**	**P8**	N	-	Y	N	Body language and/or bodily movements and/or facial expressions, and/or vocalisations	Using reinforcement

**FAM9**	**P9**	N	-	Y	N	Body language and/or bodily movements and/or facial expressions, and/or vocalisations	Communication dictionary, environmental cues to communicate transitions
**FAM9**	**P10**	N	-	Y	N	Speech (connected speech - uses sentences)	Storyboards
**FAM10**	**P11**	Y	-	Y	Y	Body language and/or bodily movements and/or facial expressions, and/or vocalisations	Baby sign language
**FAM11**	**P12**	Y	Point	Y	N	Body language and/or bodily movements and/or facial expressions, and/or vocalisations	Communication dictionary and videos/audio of behaviour, encouraging vocalisations
**FAM11**	**P13**	Beginning assessment	-	Y	N	Speech (more than 20 words)	Focussing on verbal and non-verbal communication
**FAM12**	**P14**	N	-	N	N	Body language and/or bodily movements and/or facial expressions, and/or vocalisations	Found communication board usefulness limited
**FAM13**	**P15**	Yes, does not need yet	Point	Y	N	Speech (connected speech - uses sentences)	Starting early with AAC device
**FAM13**	**P16**	N	-	Y	N	Speech (connected speech - uses sentences)	Communication partner adaptations e.g., listening intentfully
**CLN3**							
**FAM14**	**P17**	N	-	N	N	Speech (connected speech - uses sentences)	-

**FAM15**	**P18**	N	-	N	N	Speech (connected speech - uses sentences)	-
**FAM16**	**P19**	N	-	N	N	Speech (more than 20 words)	Asking closed questions, using clapping to sequence speech
**FAM17**	**P20**	N	-	Y	N	Speech (more than 20 words)	Communication partner adaptations e.g., clarifying questions. Slowing down when speaking, repeating
**FAM18**	**P21**	N	-	Y	N	Speech (connected speech - uses sentences)	Syllable timed speech
**FAM18**	**P22**	N	-	Y	N	Speech (connected speech - uses sentences)	-
**FAM19**	**P23**	Y	Partner assisted scanning	N	N	Speech (connected speech - uses sentences)	Communication partner adaptations e.g., clarifying questions, simple questions, supporting word retrieval
**FAM20**	**P24**	N	-	N	N	Body language and/or bodily movements and/or facial expressions, and/or vocalisations	-
**FAM20**	**P25**	N	-	N	N	Speech (more than 20 words)	-
**FAM21**	**P26**	N	-	N	N	Speech (connected speech - uses sentences)	Slowing down when speaking, repeating.

**FAM22**	**P27**	N	-	N	N	Speech (connected speech - uses sentences)	Communication partner adaptations e.g., speaking slowly and calmly
**FAM23**	**P28**	N	-	Y	Y	Speech (connected speech - uses sentences)	Communication partner adaptations e.g., being participant
**FAM24**	**P29**	N	-	Y	N	Speech (connected speech - uses sentences)	Communication partner adaptations e.g., being participant, extending and expanding upon single utterances
**FAM25**	**P30**	Y	-	N	N	Speech (more than 20 words)	Music therapy in combination with speech therapy, preprogrammed buttons with spoken messages, providing choices (e.g., with touch)
**FAM26**	**P31**	N	-	N	N	Speech (more than 20 words)	-
**FAM26**	**P32**	N	-	N	N	Speech (more than 20 words)	-
**FAM27**	**P33**	N	-	Y	N	Speech (connected speech - uses sentences)	-

*=Natural gesture reported as an intervention method, -=Absent/Not reported, AAC=Augmentative and alternative communication, N=No, Y=Yes
